# Plasmodial Kinase Inhibitors Targeting Malaria: Recent Developments

**DOI:** 10.3390/molecules25245949

**Published:** 2020-12-15

**Authors:** Romain Mustière, Patrice Vanelle, Nicolas Primas

**Affiliations:** Aix Marseille Univ, CNRS, ICR UMR 7273, Equipe Pharmaco-Chimie Radicalaire, Faculté de Pharmacie, 13385 Marseille CEDEX 05, France; romain.mustiere@etu.univ-amu.fr

**Keywords:** kinase inhibitor, *Plasmodium*, malaria, SAR, medicinal chemistry

## Abstract

Recent progress in reducing malaria cases and ensuing deaths is threatened by factors like mutations that induce resistance to artemisinin derivatives. Multiple drugs are currently in clinical trials for malaria treatment, including some with novel mechanisms of action. One of these, MMV390048, is a plasmodial kinase inhibitor. This review lists the recently developed molecules which target plasmodial kinases. A systematic review of the literature was performed using CAPLUS and MEDLINE databases from 2005 to 2020. It covers a total of 60 articles and describes about one hundred compounds targeting 22 plasmodial kinases. This work highlights the strong potential of compounds targeting plasmodial kinases for future drug therapies. However, the majority of the *Plasmodium* kinome remains to be explored.

## 1. Introduction

Malaria is the deadliest parasitic disease with an estimated 405,000 deaths and 213 million cases in 2018 [1], mainly in Africa. Of the estimated victims, 67% are children below five years old. The disease is caused by five *Plasmodium* species: *P. falciparum* (responsible for most of the deaths), *P. vivax* and three less important species, *P. ovale*, *P. malariae* and *P. knowlesi*. With the use of artemisinin-based combined therapies (ACT), combining an artemisinin derivate and another antimalarial drug, and vector control measures, the Word Health Organization hoped that 2020 would see a 40% reduction in malaria cases and deaths compared to 2015 [2]. However, this objective is not going to be achieved.

The vector, mosquitoes from the *Anopheles* genus, is becoming resistant to pyrethroid insecticides (used in long-lasting insecticide nets) in Africa [3]. Moreover, mutations on the *Pf*kelch13 protein, plasmodial protein recently described to be involved in hemoglobin endocytosis mechanisms [4], mediate resistance to artemisinin and its derivates. *Pf*kelch13 mutations are linked [5]:

- In vitro to a decreased susceptibility during a ring stage assay,
- In vivo to a delayed parasitic clearance.

Increased prevalence of these mutations in South-East Asia is leading to clinical failures of ACT treatments [6]. Millions of people in Africa are threatened: recent researches indicate the appearance of in vitro artemisinin resistance in Rwanda [7], and the spread of city-dwelling *A. stephensi* in Africa [8]. Thus, research and development aimed at finding new drug therapies with novel mechanisms of action is a priority. Medicines for Malaria Venture, a non-profit organization, aims to develop new antimalarial drug therapies. To guide drug discovery, target candidate profiles (TCP) have been defined for antimalarial molecules setting clear goals for new therapy [9]. There are five TCP: blood-stage killer, hypnozoites killer, hepatic schizonts killer (chemoprotection), gametocytes killer, and transmission blocker. The best compounds are the one following multiple or every TCP. One of these drug candidates, MMV390048, currently in phase II clinical trial, is the first plasmodial kinase inhibitor to reach this stage, opening the way for antimarial drugs targeting this type of protein.

Kinases are proteins catalyzing the addition of a phosphate group on a substrate, like a simple molecule or a protein. In the latter, the kinase, designed under the term of “protein kinase”, will modified the activity of the targeted protein after addition of the phosphate group. The complete set of encoded protein kinases in the genome of an organism is defined as a kinome. The human kinome consists of 518 encoded kinases [10] that play a role in the regulation of cellular processes, and whose dysregulation is involved in numerous cancers [11]. The two first human kinase inhibitors approved by the Food and Drug Administration (FDA) were rapamycin in 1999 (indicated for immunosuppression) and imatinib in 2001. Importantly, the small molecule imatinib inhibits the BCR-Abl chimeric protein, found mainly in chronic myeloid leukemia. This finding accelerated research on human kinase inhibitors and as of 1 March 2019, 48 small molecules designed as human kinase inhibitors were approved by the FDA [12], the majority of them used in oncology. According to PKIDB (a website tracking human kinase inhibitors in clinical trials), as of July 2020, 253 human kinase inhibitors were currently in clinical trials [13].

Mapping of the *P. falciparum* kinome, depending on the literature source, indicates 85 (65 for eukaryotic protein kinases (ePKs) and 20 for ePKs-related proteins) [14] or 99 [15] encoding genes, and a significant number of these proteins do not possess a human ortholog [16]. These kinases are highly conserved among the different *Plasmodium* species. Moreover, 36 plasmodial kinases (in *P. falciparum*) are identified as likely to be essential for the asexual blood-stage [17], and 15 kinases are known to be involved in *P. berghei* development in mosquitoes [18]. Thus, developing selective plasmodial kinase inhibitors could lead to multiple new antimalarial drugs with new mechanisms of action.

This review of the literature lists molecules proven to be inhibitors of plasmodial kinases and describes their properties.

## 2. Methods

This systematic review complies with the PRISMA statement defining the PRISMA checklist and PRISMA flowchart [19].

### 2.1. Data Sources and Search Parameters

Two databases were used: Scifinder (http://scifinder.cas.org, CAPLUS and MEDLINE database) and Pubmed (https://pubmed.ncbi.nlm.nih.gov/, MEDLINE database). In both databases, the keywords and Boolean operator “kinase inhibitor AND malaria” were used. The advanced search options were set to “journal” for document type and English or French for language. The research was conducted on articles published from 1 January 2005 to 1 August 2020.

### 2.2. Article Selection

Articles were selected by one reviewer, with two rounds of selection. During the first round, for each database set, articles’ titles and abstracts were assessed to eliminate irrelevant material (outside medicinal chemistry or biological fields, non-malaria related, non-filtered reviews, duplicates). To be discussed in the following review, articles had to follow the inclusion and exclusion criteria listed in Table 1.

## 3. Results

Four hundred and fifty-six articles from Scifinder (after applying the “remove duplicates” tool) and 366 articles from Pubmed were obtained. During the first round of selection, 348 Scifinder articles and 286 Pubmed articles were excluded.

The two sets were combined, and duplicates removed, leaving 121 articles. During the second round of selection, 64 articles were excluded leading to 57 selected articles; three additional articles were added in the dataset while searching for further protein information.

**Scheme 1:**
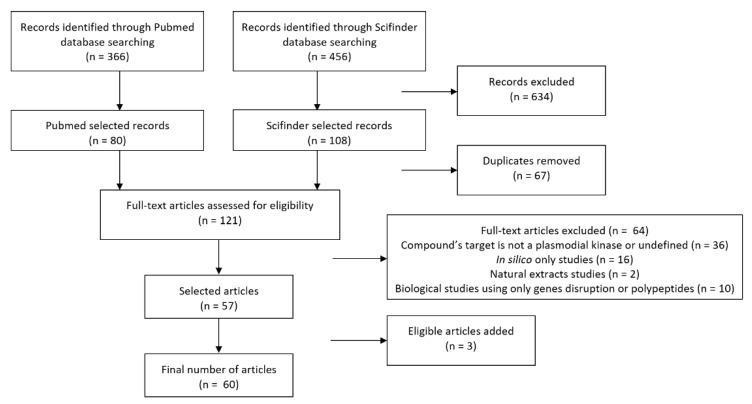
Flow diagram of article selection (derived from PRISMA flow diagram [19]).

This process is summarized in Scheme 1 in accordance with the PRISMA flow diagram [19].

## 4. Discussion

### 4.1. Molecules Targeting P. falciparum Calcium-Dependant Protein Kinases (PfCDPKs)

CDPKs can be found in plants and alveolate protists including *Apicomplexan* parasites like *T. gondii* and *Plasmodium spp* [20]. They belong to the calmodulin-dependent kinases (CaMK) family [14]. The CDPK proteins, possessing seven members (CDPK1 to CDPK7), are involved in multiple parasitic stages [21], and constitute interesting targets as they are not found in humans. Only inhibitors for *Pf*CDPK1 and *Pf*CDPK4 have been described in the literature.

#### 4.1.1. Molecules Targeting *P. falciparum* Calcium-Dependent Protein Kinase 1 (*Pf*CDPK1)

As discovered by Zhao et al. [22], *Pf*CDPK1 is involved in the invasion of red blood cells (RBC) by merozoites and their egress through activation of a protein motor complex [23,24]. More recent studies indicate the role of *Pf*CDPK1 in gametogenesis, mosquito infection [25], and regulation of *Pf*PKA (*P. falciparum* (cAMP)-dependent protein kinase) [26]. *Pf*CDPK1 was assumed to be an essential protein for *Plasmodium* since knocking out the gene during blood-stages was impossible [24]. Bansal et al., starting from a *P. falciparum* strain with a mutant *Pf*CDPK1 having reduced activity, recently managed to knock out completely *Pf*CDPK1 [25].

**Figure 1:**
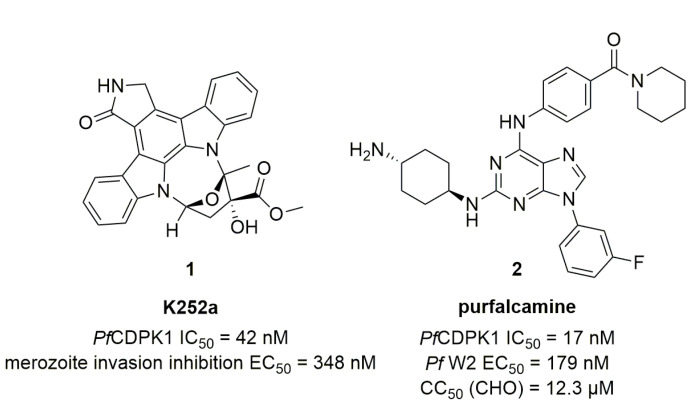
Inhibitors of *Pf*CDPK1 K252a **1** and purfalcamine **2**.

In 2008, Green et al. [23] used compound K252a (Figure 1, compound **1**) to study the motor complex regulated by *Pf*CDPK1. This indolopyrrolocarbazole displayed an IC_50_ of 45 nM toward *Pf*CDPK1 and was able to reduce RBC invasion by merozoites with an EC_50_ of 348 nM.

Kato et al. screened a library of 20,000 compounds on *Pf*CDPK1 [24]. The most active class of compounds was a series of 2,6,9-trisubstitued purines and the most potent molecule, called purfalcamine (Figure 1, compound **2**), showed an IC_50_ value of 17 nM on *Pf*CDPK1. EC_50_ in a parasitic proliferation assay on multiple *P. falciparum* strains including a multi-resistant W2 strain was determined, as well as cytotoxic values on multiple cell lines. However, the compound lacked activity in vivo in the four-day Peter’s test [27]: only a delay in onset of parasitemia was observed. Other compounds from the 2,6,9-trisubstituted purine series lacked *Pf*CDPK1 affinity or *P. falciparum* in vitro activity when the fluorine atom was replaced by a carboxylic acid or a *tert*-butoxy group. *P. falciparum* in vitro activity was also suppressed when the 4-aminocyclohexyl was replaced by a 4-hydroxycyclohexyl group.

**Figure 2:**
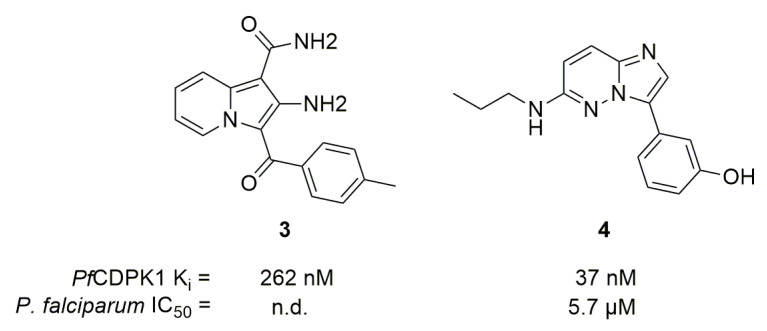
Inhibitors of *Pf*CDPK1 discovered by Lemercier et al. [28].

In 2009, Lemercier et al. identified two *Pf*CDPK1 inhibitors after a screening of 54,000 compounds [28]: the indolizine **3** and the imidazopyridazine **4** (Figure 2). **3** displayed a K_i_ of 262 nM on *Pf*CDPK1, while **4** possessed a K_i_ of 37 nM on *Pf*CDPK1 and an IC_50_ on *P. falciparum* of 5.7 µM. Out of 46 human kinases tested, compound **3** inhibited two human kinases and compound **4**, five (at 10 µM concentration). Modulation work focusing on the phenolic hydroxyl group of **4** showed a tolerance on the K_i_ value when this 3-hydroxyl was replaced with H-bond acceptors like 3,4-methylenedioxyl or 3-methoxyl, but in the latter accompanied with a 4-hydroxyl substitution.

Screening of 35,000 compounds by Chapman et al. [29] displayed imidazopyridazine as a promising series, in line with the discovery of compound **4**. An extensive Structure-Activity Relationship (SAR) work started on this scaffold (Table 2). Chapman et al. [30] explored the side chains at positions 3 and 6 of the imidazopyridazine core. Work on the R_1_ substituent largely targeted the introduction of cyclic amines, while work on R_2_ addressed *para*- or *meta*-functionalized phenyls, pyridines, or pyrimidines. Compound **5** (Table 2) displayed interesting results with an IC_50_ on *Pf*CDPK1 of 13 nM, an IC_50_ on *Pf*3D7 of 400 nM, and the ability to reduce by 46% the parasitemia, in a *P. berghei* mouse model *per os* during a four-day Peter’s test. Finally, **5** showed inhibition toward 12 human kinases (out of 73 tested) ranging between 50% and 80% at 1 µM.

Large et al. continued this work with the introduction of *N*-substituted imidazoles at position 3 of the imidazopyridazine core while modulating position 6 with substituents like aminocyclohexyl or *N*-methylpiperidine [31]. This resulted in the synthesis of compound **6** (Table 2) showing an improved activity on *Pf*3D7 compared to compound **5** and improved ligand-lipophilicity efficiency (LLE) with a score of 6, compared to 4.5 for compound **5**. Inhibition assays on human kinases were not performed on compound **6**, but other compounds from this series showed lower selectivity than compound **5**.

The last work on this series was done by Chapman et al. [32] using docking studies on the T. gondii CDPK1 ortholog structure to guide them. Starting from **5**, they started by modulating R_2_ with N-substituted 2-aminopyrimidines, hoping for improved in vivo results. Compound **7** (Table 2) emerged as the best compound with improved in vitro parameters. However, low PAMPA (parallel artificial membrane permeability assay) permeability (4 P_app_/nms^−1^) led to poor results in vivo, with only 4% reduction of parasitemia in the four-day Peter’s test [27]. Out of 66 human kinases tested at 1 µM concentration, **7** inhibited nine human kinases by at least 80%. Additional work was then done: the side chain at position 6 was modulated with different cyclic amines, and at position 3, the fluorine atom position on both cycles was explored along with the nature of the two cycles (phenyls or pyridines). This led to compound **8** (Table 2) with slightly lower activity in vitro than **7**. The reduction of parasitaemia in P. berghei mouse model was slightly better at 51% than that of **5** at 46% (Table 2).

**Figure 3:**
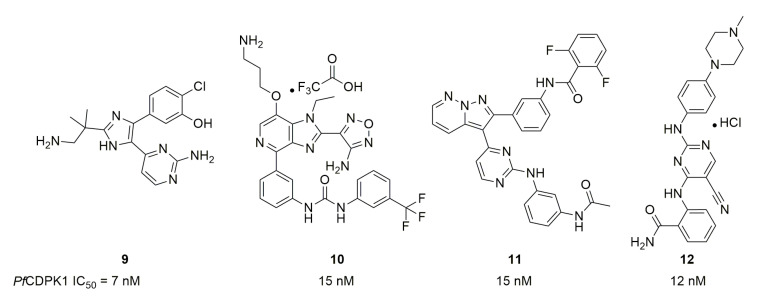
Best compounds, from each chemical series having a molecule with an IC_50_ equal to or below 20 nM toward *Pf*CDPK1 in the screening realized by Crowther et al. [33].

In 2016, Crowther et al. screened 14,000 compounds on multiple plasmodial kinases including *Pf*CDPK1 [33]. One hundred and eighty-one molecules showed a sub-micromolar IC_50_ on *Pf*CDPK1 and 12 compounds grouped in four chemical series displayed an IC_50_ equal to or below 20 nM. The best compounds from each series are represented in Figure 3.

#### 4.1.2. Molecules Targeting *Plasmodium falciparum* Calcium-Dependent Protein Kinase 4 (*Pf*CDPK4)

*Pf*CDPK4 is a key enzyme for the exflagellation of gametocytes, a mechanism leading to the formation of microgametocytes, the male gametocytes, in the mosquito midgut [34,35,36].

**Figure 4:**
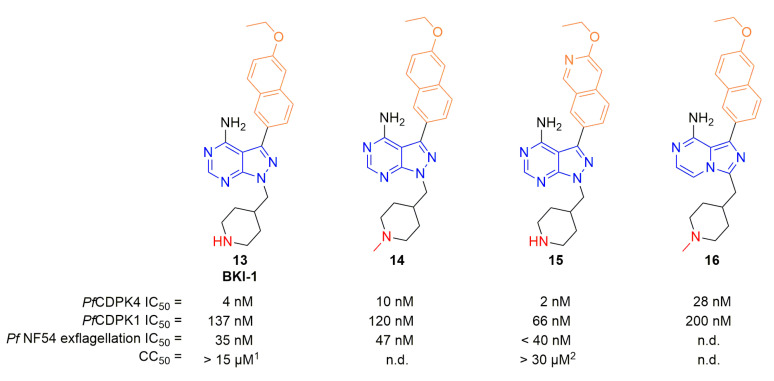
4-Aminopyrazolopyrimidine and 4-aminoimidazopyrazine inhibitors of *Pf*CDPK4. ^1^ On mammalian fibroblast cells. ^2^ On HepG2 cells.

In 2012, Ojo et al. described the properties of BKI-1 (compound **13**), a disubstituted pyrazolo[3,4-*d*]pyrimidin-4-amine [36] (Figure 4). **13** displayed an IC_50_ on *Pf*CDPK4 of 4.1 nM, an IC_50_ on *Pf*CDPK1 of 136 nM, an EC_50_ toward asexual stages of 2 µM (unsurprisingly since CDPK4 is important in the sexual stage) and was able to inhibit *P. falciparum* exflagellation with an EC_50_ of 35 nM. **13′**s selectivity was assessed with IC_50_ measures on *Hs*ABL and *Hs*SRC, two human kinases, who were superior to 50 and 20 µM, respectively. Lastly, on a *P. berghei*-infected mouse model, **13** was able to completely inhibit the formation of oocysts in mosquitoes at the intraperitoneal dose of 10 mg/kg.

Ojo et al. then carried out minor pharmacomodulations on **13** [37] including the replacement of the piperidine with a *N*-methylpiperidine, morpholine or pyrane heterocycle, replacement of the naphthalene by a quinoline and replacement of the methoxy group by an ethoxy group. Activities on the enzyme and the parasite were maintained for all molecules except the quinoline analog without a methoxy group. Compound **14**, the *N*-methypiperidine analog of **13** (Figure 4) showed similar in vitro activity parameters to **13** but clear improvements in in vitro and in vivo absorption-distribution-metabolism-elimination-toxicology (ADMET) parameters (Table 3). Selectivity against human kinases was conserved as only one protein out of the 80 assessed was found to be inhibited. The only downside to **14** was its hERG channel activity, which was reduced, from 0.767 µM to more than 10 µM, by replacing the *N*-methylpiperidine with a pyrane.

This work was followed by a massive medicinal chemistry study by Vidadala et al. focused on the modulation of the lateral chains of the pyrazolo[3,4-*d*]pyrimidin-4-amine core [38]. ATP binding sites of both *Pf*CDPK1 and *Pf*CDPK4 are very similar to the one found in *T. gondii* CDPK1. Thus *Tg*CDPK1 was used for docking studies with previously described *Pf*CDPK4 inhibitors to guide pharmacomodulations. The C_3_ side-chain was the first to be modulated while keeping an isopropyl or *tert*-butyl group as *N*-substituent. The best C_3_ group was then used during the modulation of the *N*-substituent. A scaffold hopping strategy was also carried out, changing the pyrazolopyrimidine to an imidazopyrazine core. This led to compound **15** with an interesting dual activity on *Pf*CDPK1 and *Pf*CDPK4 (Figure 4) and a conserved selectivity versus *Hs*SRC (IC_50_ > 10 µM). The scaffold hopping strategy was successful, as illustrated by compound **16**. The authors stated that they are now seeking to enhance the metabolic stability of their compounds in vivo, since they need to remain in the bloodstream for a period of three to four weeks to have their effect on gametocytes.

**Figure 5:**
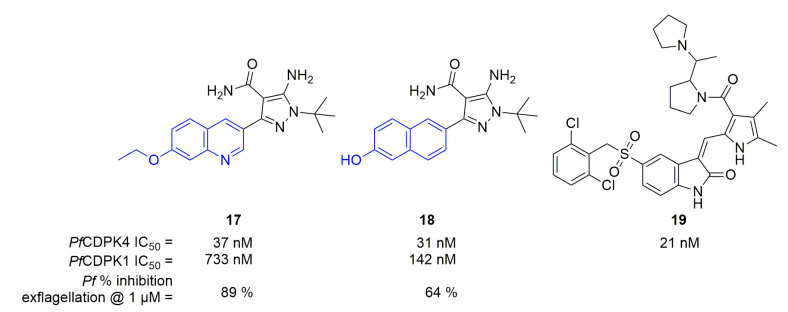
Aminopyrazole-carboxamide and indolone inhibitors of *Pf*CDPK4.

In 2016, Huang et al. used on 5-aminopyrazole-4-carboxamides, known to work on *Toxoplasma gondii* and *Cryptosporidium parvum* CDPK1 (orthologs of *Pf*CDPK4), to obtain new *Pf*CDPK4 inhibitors [39]. They synthesized 28 compounds; most of which have IC_50_ toward *Pf*CDPK4 below 100 nM. The best compound regarding *Pf*CDPK4 inhibition was **17**, while **18** was interesting with a dual *Pf*CDPK1/4 inhibition (Figure 5). These two compounds did not possess any activity on hERG channels. **17** was selective as it possessed an IC_50_ superior to 30 µM against *Hs*SRC. Work on this series is currently focused on *C. parvum* therapy [40].

In the Crowther et al. screening [33], 55 compounds showed an activity below the micromolar range against *Pf*CDPK4. The best compound (**19**) displayed an IC_50_ of 21 nM (Figure 5).

The only thing that prevents *Pf*CDPK4 inhibitors from entering more advanced pre-clinical studies is the long-term bioavailability they would need to be active against gametocytes. Moreover, finding from Vidadala et al. [38] and data from screening by Crowther et al. [33], identified compounds providing dual inhibition of *Pf*CDPK1 and *Pf*CPDK4, offering the prospect of compounds with action on two stages out of three of the *Plasmodium* cycle.

### 4.2. Molecules Targeting Plasmodium falciparum Choline Kinase (PfCK)

Choline kinase is an enzyme involved in the synthesis of phosphatidylcholine; phospholipid most frequently found in *P. falciparum*. It transforms choline into phosphocholine, and is also involved in the synthesis of phosphatidylethanolamine, the second most common phospholipid [41]. Inhibition of *Pf*CK has been shown to reduce parasite growth in vitro [42,43]. The active sites of *Pf*CK have 69% similarity with the human enzyme *Hs*CKα1 [44].

**Figure 6:**
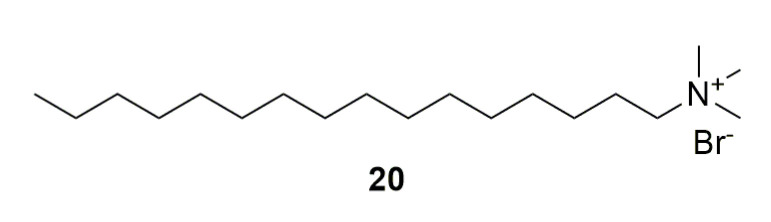
Hexadecyltrimethylammonium bromide **20** structure.

In 2007, Choubey et al. [45] studied hexadecyltrimethylammonium bromide (compound **20**, Figure 6). It displayed in vitro, at 10 µM and 20 µM, parasitic growth inhibition of 62% and 81%, and reduced phosphocholine synthesis by 57% at 10 µM. In a *P. yoelii* mouse model, it was able to reduce parasitemia by almost 50% after four days of intravenous doses of 5 mg/kg.

**Figure 7:**
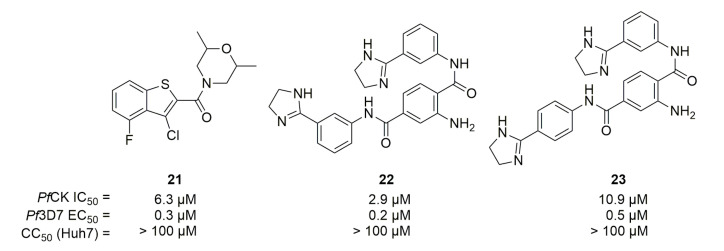
Hits from Crowther et al. against *Pf*CK [46].

A screening by Crowther et al. on around 5000 commercially available small molecules led to the discovery of three hits against *Pf*CK [46] (compounds **21**, **22** and **23**, Figure 7).

While studying *Pf*CK metabolic activities, Serrán-Aguilera et al. used two compounds (**24** and **25**, Table 4) to inhibit the enzyme [41], which possessed a K_i_ between 30 and 40 µM (when assessed for choline conversion to phosphocholine). To explore the activity of these pyridinium salts on *Pf*CK, Schiafino-Ortega et al. assessed 1,2-diphenoxyethane salts with a symmetrical structure [47] close to **24** and **25**. Bisquinolinium bromide salts derivatives were the most potent with submicromolar activity on *P. falciparum*. However, their activity on the *Pf*CK enzyme was found to be above 2 µM for all this series, raising doubts about their real mechanism of action. The best compound of this series was compound **26**, with an improved *Pf*CK_Cho_ IC_50_ (formation of phosphocholine is measured) reduced from 103 to 2.4 µM, but it no longer showed *P. falciparum* in vitro activity (Table 4). Compound **26** was not selective as it also inhibited the human *Hs*CKα1 enzyme.

Most of the molecules presented above (from **21** to **25**) probably target more than just *Pf*CK, as enzyme IC_50_ values not in line IC_50_ on the parasite: **24** is the best example of such behavior. Furthermore, the design of these compounds needs to take into account the human choline kinase. Thus, *Pf*CK inhibitors are potentially valuable compounds, they are still in the early stages of development and promising hits have yet to be discovered.

### 4.3. Molecules Targeting Plasmodium falciparum Casein Kinase 2 (PfCK2)

Casein kinases are serine/threonine protein kinases found in eukaryotic organisms. Two casein kinases can be found in *P. falciparum*: CK1 and CK2. *Pf*CK2 is thought to be a key enzyme during the asexual blood-stage of the parasite. It has been shown that many proteins are possibly phosphorylated, and thus have their activity regulated, via *Pf*CK2 during the asexual blood-stage [48]. Both *Pf*CK1 and *Pf*CK2 have been demonstrated to be essential for the asexual blood-stage [17,49].

**Figure 8:**
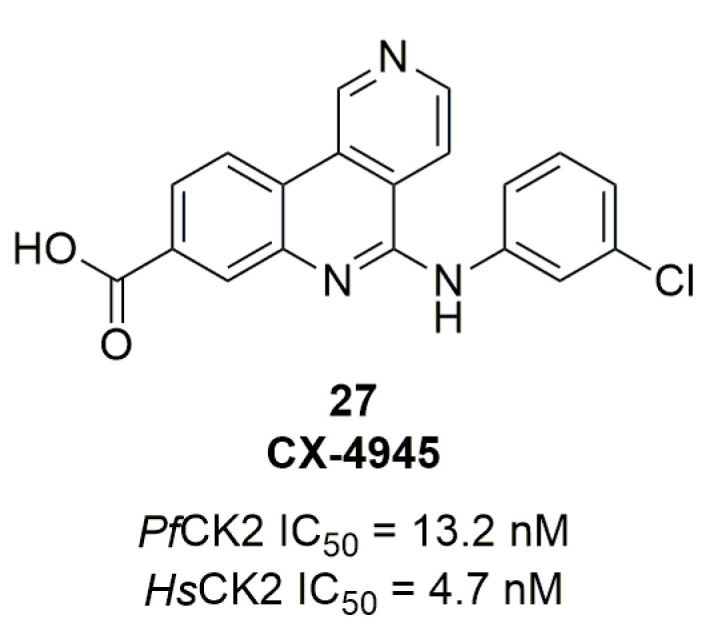
CX-4945, an *Hs*CK2 inhibitor in clinical trials, also inhibits *Pf*CK2.

While studying the *Pf*CK2 catalytic domain, Ruiz-Carillo et al. used CX-4945 (Figure 8, compound **27**) [50], a human CK2 inhibitor currently in multiple phase II clinical trials in oncology [51], to inhibit *Pf*CK2 with an IC_50_ of 13.2 nM.

### 4.4. Molecules Targeting Plasmodium falciparum Cyclin-Dependent-Like Kinase (PfCLK)

The CLK family includes four enzymes (*Pf*CLK1 to *Pf*CLK 4) involved in the phosphorylation (and the activity modulation) mainly of serine-arginine-rich proteins found in spliceosomes [14,52,53]. Spliceosomes are complexes of proteins involved in the removal of introns in pre-messenger RNA. All *Pf*CLKs are considered essential for the asexual blood-stage [17]. Inhibition of CLKs was first linked to a schizonticide and gametocytocidal effect [53]. A more recent study on *Pf*CLK3 showed that inhibition of this protein affected all three stages of the malaria cycle [54].

In 2014, Kern et al. studied the effect of inhibiting *Pf*CLKs [53]. The authors did not manage to disrupt the genes directly and therefore turned to chemical compounds. They screened 63 *Hs*CLK inhibitors against *Pf*CLKs. Two compounds, **28** and chlorhexidine (**29**) showed interesting results regarding *Pf*CLK inhibition and *Pf*3D7 growth inhibition; results are summarized in Table 5.

**Figure 9:**
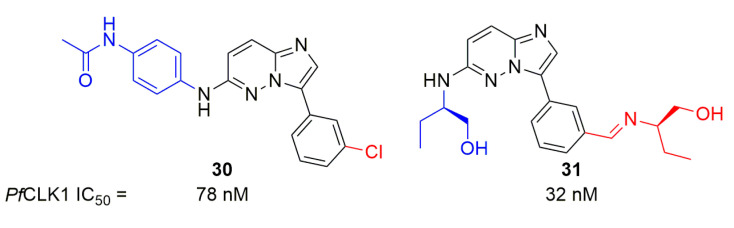
Imidazopyridazines inhibitors of *Pf*CLK1.

In 2017, Bendjeddou et al. tested an imidazopyridazine series on multiple human and parasitic kinases, including *Pf*CLK1 [55]. Two compounds (**30** and **31**, Figure 9) showed activity on *Pf*CLK1 below 100 nM but lacked selectivity as they also targeted human kinases, in some cases with IC_50_ below 100 nM.

**Figure 10:**
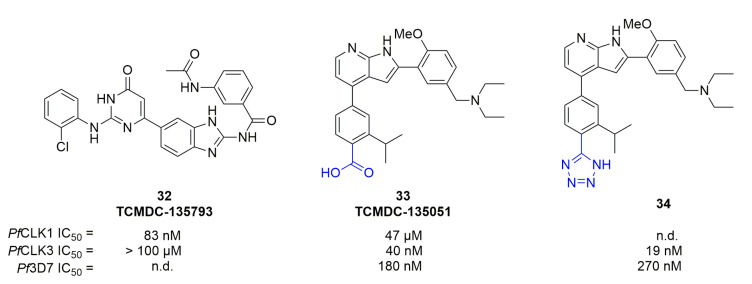
Molecules inhibiting *Pf*CLK1 or *Pf*CLK3.

More recently, Alam et al. screened 30,000 compounds on *Pf*CLK1 and *Pf*CLK3 [54]. TCMDC-135793 (compound **32**) and TCMDC-135051 (compound **33**) were among the most potent and selective compounds on *Pf*CLK1 and *Pf*CLK3, respectively (Figure 10). Target was confirmed for **33** with *Pf*Dd2 strain possessing a mutant *Pf*CLK3 that showed an increased IC_50_ value. Compund **33** was active at all stages, inhibiting:

- Schizont development in blood-stage,
- Sporozoite invasion and development in the liver stage,
- Gametocyte development,
- Exflagellation in the mosquito midgut.

SAR work associated with **33** was carried out by Mahindra et al. [56]. Modulations targeting the substituents of the phenyl ring at position 2 of the pyrrolo[2,3-b]pyridine core led to analogs possessing an IC_50_ against *Pf*CLK3 below 100 nM. However, micromolar activity was found against *Pf*3D7 when the diethylamine was changed to dimethylamine, primary amine or morpholine substituents. The position of the methoxy substituent appeared to be important for activity. Similar modifications were carried out on the other phenyl ring: removing the isopropyl substituent or replacing it by methyl led to compounds with micromolar activities against *Pf*3D7, and removing the carboxylic acid or changing it to an ethyl ester resulted in coumpounds with micromolar activity on *Pf*CLK3. Replacing the carboxylic acid to a tetrazole, a known bioisoster, led to compound **34** showing a slight improvement in *Pf*CLK3 activity, with an IC_50_ of 19 nM but with an increased *Pf*3D7 IC_50_ to 270 nM. **34** stability was conserved in vitro in mouse liver microsomes with an intrinsic clearance at 2.32 mL/min/g of liver, compared to **33** with 1.33.

### 4.5. Molecules Targeting Coenzyme A Synthesis Pathway Kinases: Plasmodium falciparum Pantothenate Kinase and Dephospho-Coenzyme A Kinase (PfPanK & PfDPCK)

Coenzyme A is a molecule found in many metabolic pathways in eukaryotic life forms. In *P. falciparum*, coenzyme A is synthesized in five steps starting from pantothenate; two of these steps involve kinases:

- the first step, transforming pantothenate into 4′-phosphopantothenate, is catalyzed by *Pf*PanK,
- the last step, transforming dephospho-coenzyme A into coenzyme A, is catalyzed by *Pf*DPCK.

While the synthesis and the function of coenzyme A are well preserved around all organisms that possess it [57], the enzymes’ coding sequences can differ.

Studies on the inhibition of *Pf*PanK focused on the synthesis of pantothenate (Table 6, compound **35**) analogs able to competitively inhibit 4′-phosphopantothenate synthesis. Spry et al. synthesized ten analogs with various chains on the nitrogen atom of the amide group [58]. Compound **36** was the best of the series, with an IC_50_ of 76 µM on *Pf*3D7 (at 1 µM pantothenate concentration). Increasing the pantothenate concentration increased this IC_50_ suggesting a mechanism of action related to the pantothenate metabolism. Changing the terminal group of the chain or its length did not affect the activity. Spry et al. recently investigated the synthesis of analogs of CJ-15,801 (**37**) [59], a *trans* enamide analog of pantothenate. Modulations were focused on the ester and modifying the diol side chain. Compound **38** showed an improved IC_50_ of 13 µM on *Pf*3D7 compared to 36 µM for **37** but most importantly a 100-fold decreased of the *Pf*PanK IC_50_ value. In 2017, Chiu et al. described compound **39** as a pantothenate kinase inhibitor with an IC_50_ of 30 nM on *Pf*W2 [60]. Adding more pantothenate to the growing medium increased this value. The authors also demonstrated that **39** was able to inhibit the *Pf*PanK homolog of *S. cervisiae*, Cab1.

**Figure 11:**
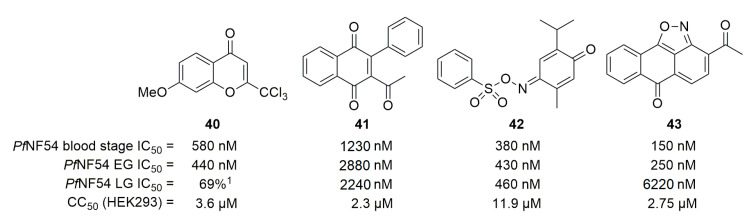
Inhibitors of *Pf*DPCK from Fletcher et al. [61]. ^1^ At 40 µM. EG = Early-stage Gametocytes, LG = Late-stage Gametocytes.

Concerning *Pf*DPCK, work by Fletcher et al. identified some potent compounds [61]. The activity of assessed compounds on *P. falciparum* was counterbalanced by adding coenzyme A to the culture medium to identified compounds active on coenzyme A synthesis. Similar rescue tests were then done with *Pf*PanK or *Pf*DPCK substrates. Four compounds (**40**–**43**, Figure 11) showed possible activity on *Pf*DPCK, affecting the blood-stage and gametocytes.

### 4.6. Molecules Targeting Plasmodium falciparum FIKK Kinases (PfFKks)

FIKK kinases are named after a shared sequence of four amino acids (Phe-Ile-Lys-Lys or FIKK) and are an important group of kinases in malaria parasites [14]. While *P. vivax* and *P. berghei* possess one FIKK kinase, *P. falciparum* differs completely, possessing 20 FIKK kinases. The FIKK kinase gene may be essential for the parasite [18], yet the role of these proteins remains unclear. *Pf*FKks are exported into the RBC cytosol (except *Pf*FKk8), where they possibly interact with RBC’s cytoskeletal proteins and membrane [62,63].

**Figure 12:**
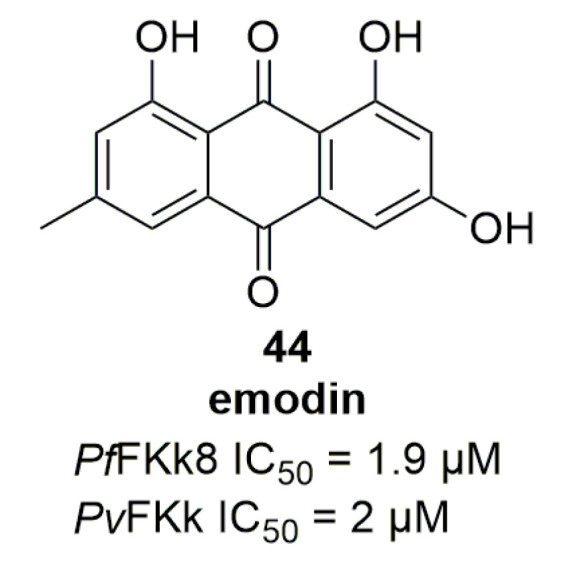
Chemical structure of emodin.

The only studies on inhibitors of FKks were performed by Lin et al. [64,65], who screened compounds on *Pf*FKk8 and *P. vivax* FKk (catalytic domain only for the latter). In both articles, emodin (**44**, Figure 12), an anthraquinone, showed an IC_50_ of 1.9 µM and 2 µM against these tested proteins, respectively.

### 4.7. Molecules Targeting Plasmodium falciparum Guanylate Kinase (PfGK)

**Figure 13:**
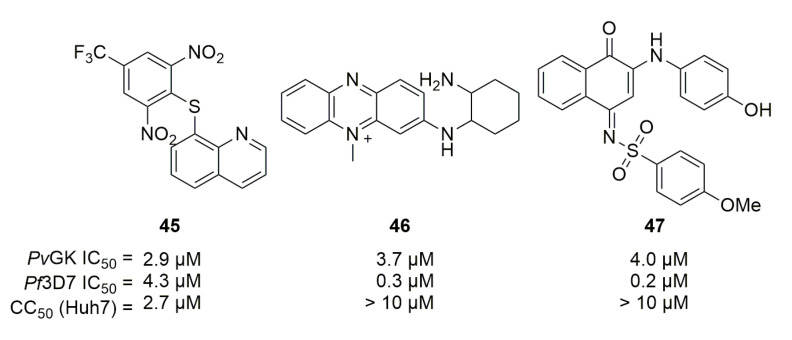
Inhibitors of *Pv*GK.

Guanylate kinase catalyzes the transformation of (deoxy)guanosine-monophosphate into (deoxy)guanosine-diphosphate. However, this transformation can potentially be bypassed by *P. falciparum* thymidylate kinase, which can also catalyze the *Pf*GK reaction [66]. In their screening [46], Crowther et al. included guanylate kinase as one of their screened proteins but used *Pv*GK (possibly because guanylate kinase expression is greater at liver stage [67]). Three compounds emerged as potential hits (**45** to **47**, Figure 13), but as the authors stated, structural features do not currently make them good candidates for further medicinal chemistry studies.

### 4.8. Molecules Targeting Plasmodium falciparum Glycogen Synthase Kinase 3 (PfGSK3)

*Pf*GSK3 is possibly an essential enzyme for the parasite asexual blood-stage [17] but its implication in biological pathways is not clear. One of its phosphorylation targets, the apical membrane antigen 1 (AMA1) [68], is required for RBC invasion by merozoites. *Pf*GSK-3 possesses a human ortholog, *Hs*GSK3. *Hs*GSK3 hyperactivation is linked to Alzheimer’s disease, diabetes, or cancers and many compounds inhibiting *Hs*GSK3 have been described [69].

In 2013, Fugel et al. realized a high-throughput screening of 10,000 molecules on *Pf*GSK3 [70]. Multiple molecules with a thieno[2,3-b]pyridine core were found to be hits against *Pf*GSK3. Forty-three analogs from four different thieno[2,3-b]pyridines scaffolds were synthesized. The 3,6-diamino-4-(2-halophenyl)-2-benzoylthieno[2,3-b]pyridine-5-nitriles showed the best results on *Pf*GSK3 and *Sus scrofa* GSK3 (used for selectivity comparison). *Hs*GSK3 IC_50_ and *Pf*NF54LUC EC_50_ tests were done on the best compound, **48** (Table 7).

Based on a docking study of **48** on an analogy model of *Pf*GSK3, Masch el al. synthesized 23 analogs of **48** with different substituents (halogens, ethers, alkylated amines, cyclic amines) at position 4 of the 2-chlorophenyl cycle [71] (Table 7). Compound **49** showed lower activity on *Pf*GSK3 but its potency on in vitro parasites was improved compared to **48**. Aqueous solubility was improved, going from 1.5 µM for **48** to 4.8 µM for **49**. The authors explored the possible axial chirality created by the 2-chlorophenyl cycle. They isolated the two isomers of **50** and compared their activity: (+)-**50** was active but not (−)-**50**. According to the authors, this difference arose from the chlorine atom position in (−)-**50**, which prevented it from fitting into the protein binding pocket.

### 4.9. Molecules Targeting Plasmodium falciparum Hexokinase (PfHK)

Hexokinase is the first enzyme implicated in glycolysis, catalyzing the transformation of glucose into glucose 6-phosphate. Studies showed that *Pf*HK inhibition (directly or indirectly by inhibiting glucose transporters) is linked to parasite death during the asexual blood-stage [72,73]. *Pf*HK only possesses 24% similarity with human glucokinase, suggesting the possibility of designing selective *Pf*HK inhibitors [74].

In 2013, Harris et al. screened a small library of compounds on *Pf*HK [74]: three simple benzoisothiazolinones (**51**–**53**, Table 8) and the closely-related selenium compound ebselen (**54**) showed activity against *Pf*HK below 300 nM. These values were not completely correlated regarding EC_50_ against *Pf*3D7, as stated by the authors, who suggested that cell permeability and off-target interaction problems might explain the difference.

In another screening from the same team, Davis et al. screened 50,000 compounds on *Pf*HK [75]: two compounds (**55** and **56**, Table 9) displayed a micromolar activity on *Pf*HK but lacked activity on *Pf*3D7 in vitro.

### 4.10. Molecules Targeting Plasmodium falciparum Mitogen-Activated Protein Kinase 2 (PfMAP2)

**Figure 14:**
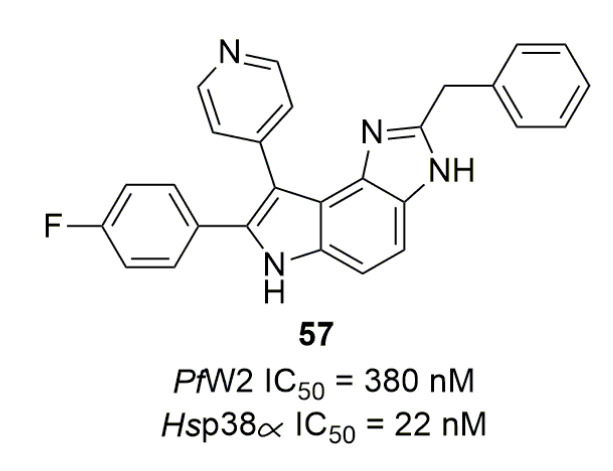
Inhibitor of *Pf*map-2 discovered by Brumlik et al. [77].

Mitogen-activated protein kinases (MAPK) are proteins involved in signal transduction and are the key to many cellular processes in eukaryotic organisms. Two MAPKs are found in *P. falciparum*: *Pf*MAP1 and *Pf*MAP2. Very recently, Hitz et al. defined the role of these two proteins: neither is essential for asexual development, but *Pf*MAP2 is essential for exflagellation, a mechanism that appears to be directly linked to this protein without the involvement of *Pf*MAP1 [76]. Brumlik et al. screened some *Hs*p38α (a human MAPK) inhibitors to assess their activity on *P. falciparum* [77]. One compound (**57**) displayed sub-micromolar activity on *Pf*W2 (Figure 14).

### 4.11. Molecules Targeting Plasmodium falciparum MO15-Related Protein Kinase (PfMRK)

*Pf*MRK is a cyclin-dependent kinase located in the nucleus. It interacts with two other proteins [78]: CDK-activating kinase assembly factor (*Pf*MAT1) and cyclin 1 (*Pf*cyc-1). These three proteins are considered likely to be important for gene expression and DNA replication, and *Pf*MRK is believed to be essential for the asexual blood-stage [17]. *Pf*MRK shares the most properties, regarding human proteins, with *Hs*CDK7.

**Figure 15:**
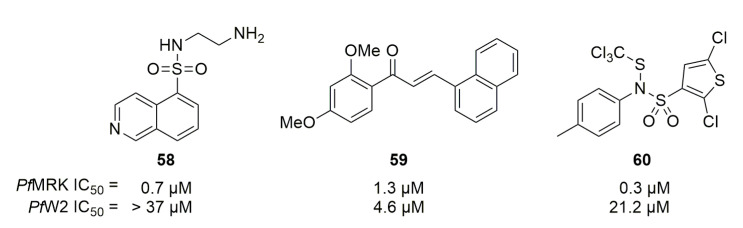
Inhibitors of *Pf*MRK.

Woodard et al. screened 12 isoquinoline and naphthalene sulfonamides, known for being kinase inhibitors, on *Pf*MRK [79]. Only **58** displayed a sub-micromolar activity on *Pf*MRK but was not active on *P. falciparum* in vitro. The same team later designed some chalcones based on an in-silico model of *Hs*CDK7 [80]. One of these chalcones, **59**, displayed a 1.3 µM activity against *Pf*MRK associated with an in vitro activity of 4.6 µM on *Pf*W2. Caridha et al. tested 27 sulfonamide compounds substituted by phenyl or thiophene cycles [81]. Ten molecules, including compound **60** (Figure 15), possessed a sub-micromolar activity on *Pf*MRK, but none was active on *Pf*W2. Cytotoxicity of compound **60** on five different cell lines was assessed, and all the values were around or above 25 µM.

### 4.12. Molecules Targeting Plasmodium falciparum NIMA-Related Kinase 1 (PfNEK1)

Never-in-mitosis/Aspergillus (NIMA) related protein kinases are found in multiple eukaryotic organisms and are involved in the cell division process. Four NEK proteins have been discovered in *Plasmodium* [82]:

- *Pf*NEK2 and *Pf*NEK4 are related to sexual stages of the parasite,
- *Pf*NEK1 and *Pf*NEK3 can interact with *Pf*MAP2 and,
- *Pf*NEK1 is also expressed during schizogony (it is the only NEK essential in the blood-stage) and in male gametocytes.

**Figure 16:**
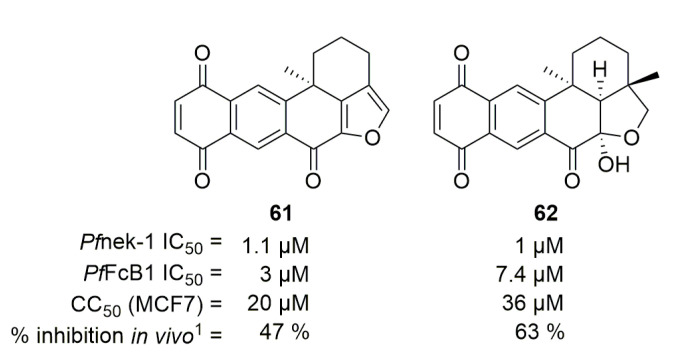
Natural quinone inhibitors of *Pf*nek-1. ^1^ 4-day Peter’s test at 5 mg/kg/4 days intraperitoneal.

Laurent et al. and Desoubzdanne et al. found two natural compounds targeting this protein (Figure 16): xestoquinone **61** and alisiaquinone **62** [83,84] displayed interesting in vitro parameters and an in vivo activity on a mouse model at 5 mg/kg during a four-day Peter’s test [27]. Xestoquinone **61** was found to be selective of *Pf*NEK1 versus other plasmodial and human kinases.

### 4.13. Molecules Targeting Plasmodium falciparum Phosphatidyl-Inositol 4-Kinase (PfPI4K)

Phosphatidyl-inositol 4-kinase catalyzes the transformation of phosphatidyl-inositol into phosphatidyl-inositol 4 phosphate (PI4P). PI4P is a key secondary messenger involved in multiple cellular pathways. Inhibition of *Pf*PI4K led to activity on every stage of the *Plasmodium* cycle [85] with inhibition of:

- Development of schizonts and hypnozoites in the liver stage,
- Asexual growth and gametocytogenesis in blood-stage,
- Reduction of oocysts in mosquito midgut in the sexual stage.

*Pf*PI4K inhibition phenotypic consequences were found by McNamara et al. using four compounds as tools (three are showed in Table 10) [85]. The most assessed compound, KDU691 (**63**) an imidazopyrazine, showed promising in vivo activity on luciferase-expressing *P. berghei*-infected mice: with a single dose of 7.5 mg/kg, **63** was able to protect mice from what would be otherwise a fatal infection. **63** is selective of *Pf*PI4K versus other human kinases including *Hs*PI4K (IC_50_ = 7.9 µM). Other tested compounds included the imidazopyridazine KAI715 (**64**) and the quinoxaline BQR695 (**65**). Their in vitro results are summed up in Table 10. *Pf*PI4K was confirmed as the target of these compounds by analysis of resistant clones and artificial modifications of *Pf*PI4K gene were also associated with drug resistance.

**Figure 17:**
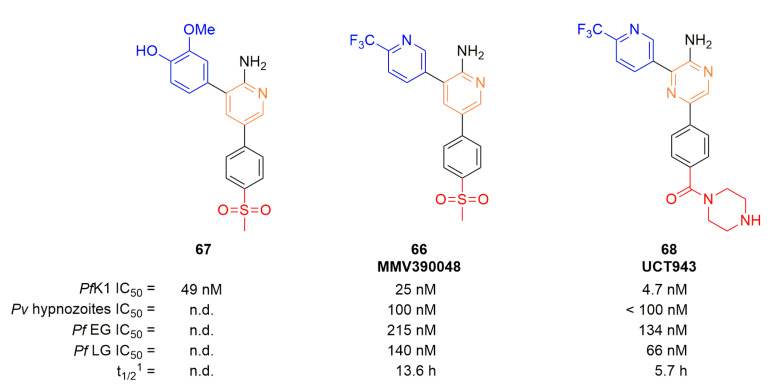
Starting from **67**, medicinal chemistry work led to **66** which was further improved to **68**. EG = Early-stage gametocytes, LG = Late-stage gametocytes. ^1^ mouse model, 20 mg/kg per os.

*Pf*PI4K inhibitors are the most advanced plasmodial kinase inhibitors in terms of drug development. One compound, MMV390048 (**66**), is currently in phase II of clinical trials and is the only plasmodial kinase inhibitor currently in clinical trial (Figure 17) [86]. MMV390048 PI4K inhibitory effects, with the consequence of a multistage activity, were demonstrated by Paquet et al. using chemoproteomics studies [87]. This inhibition is selective, as *Hs*PI4Kα and *Hs*PI4Kβ were not targeted by **66**. Numerous in vivo experiments were performed (on mice, rats, dogs, and monkeys): multistage potency was conserved in in vivo models and **66** displayed very good pharmacokinetic parameters including bioavailability of 74% and a half-time of 66 h at an oral dose of 5 mg/kg on monkeys. All these parameters pointed **66** as a strong lead compound candidate for clinical studies for single exposure radical cure; it could also be promising for chemoprevention. Compound **66** originally emerged from a SAR study by Younis et al. which, starting from compound **67**, focused on modulation of the methoxyphenol group [88].

Initial human clinical studies on **66** “showed high variability in exposure, which was attributed to low aqueous solubility” [89]. To solve, this problem, Brunshwig et al., synthesized UCT943 (**68**), an analog of **66** where the methylsulfone was replaced by a piperazinyl carboxamide and the aminopyridine by an aminopyrazine to improve aqueous solubility [89]. This second generation of *Pf*PI4K inhibitor showed improved parameters on every stage of the malaria cycle. ADMET properties were impacted by the modification but the in vivo activity was conserved.

**Figure 18:**
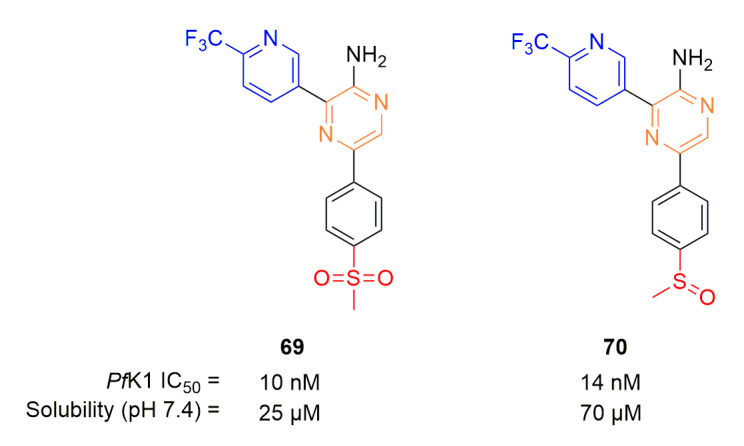
Aminopyrazines derivatives used to study a sulfoxide-to sulfone produg approach.

Continuing on this new aminopyrazine scaffold, Gibhard et al., synthesized two aminopyrazines (Figure 18) to explore a potential prodrug strategy by conversion of a sulfoxide into a sulfone [90]. Compounds **69** (pyrazine analog of **66**) and **70** showed nanomolar activities in vitro. Solubility was better at physiological pH for the sulfoxide derivative compared to the sulfone. In vivo, the 90% effective dose (ED_90_) per os was at 0.12 mg.kg^−1^ for both compounds compared to 0.57 mg.kg^−1^ for **66** and 0.25 mg.kg^-1^ for **68** (on *Pf*3D7 infected mouse model). As expected by the authors, **70** was found to be rapidly converted in vivo into **69**, validating the sulfoxide prodrug approach for this series of compounds.

**Figure 19:**
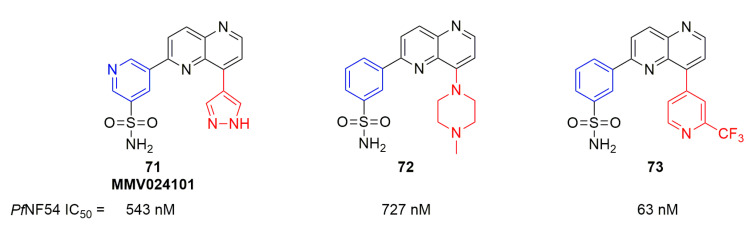
1,5-naphthyridines as inhibitors of *Pf*PI4K.

Other compounds with original scaffolds have been discovered since the development of **66**. Kandepedu et al. described a series of 1,5-naphthyridines, most of them having submicromolar activities on *Pf*NF54 [91]. Starting from MMV024101 (**71**), a SAR study was performed on the two substituents of the naphthyridine core (Figure 19) and 48 analogs were synthesized. Compound **73** showed good activity on *Pf*NF54 with an IC_50_ of 63 nM and good metabolic stability (t_1/2_ = 33 h at 5 mg/kg per os) but limited oral bioavailability (39%) on a mouse model.

**Figure 20:**
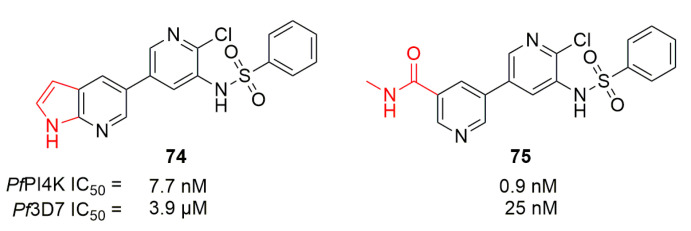
2-chloro-3-sulfonamide pyridines as inhibitors of *Pf*PI4K.

Liang et al. described a bipyridine series targeting *Pf*PI4K from screening in their compound library [92]. Compound **74** displayed an activity around 4 µM on *Pf*3D7 but was able to inhibit *Pf*PI4K with an IC_50_ of 7.7 nM (Figure 20). An in-silico homology model of *Pf*PI4K was then used to rationally guide modulations on **74**: the 2-chloro-3-sulfonamide pyridine core was kept and modulations were performed on the two side chains. Compound **75** displayed the best activity on *Pf*3D7 of 25.1 nM together with an IC_50_ on *Pf*PI4K of 0.9 nM. This activity was shown to be selective of *Pf*PI4K versus human kinases including *Hs*PI3K or *Hs*PI4K. With oral bioavailability of 80% and a t_1/2_ of 3.6 h on a mouse model (at 10 mg/kg per os), **75** was able in vivo to cure blood-stage *P. yoelii* at 80 mg/kg/7 days and to stop liver-stage infection by *P. berghei* sporozoites at a single dose of 1 mg/kg in a mouse model.

### 4.14. Molecules Targeting Plasmodium falciparum Protein Kinase 5, 6, 7 and 9 (PfPK5, PfPK6, PfPK7 and PfPK9)

*Pf*PK5 is a cyclin-dependent-like kinase [93], essential for the parasite blood-stage [17], with functions still unclear. Because of its homology with *Hs*CDK2 [94], *Pf*PK5 could be linked to cell division regulation [95]. *Pf*PK6 is another cyclin-dependent like kinase most likely essential for the asexual blood-stage [17,18,96]. *Pf*PK7 is an orphan kinase (it does not cluster with defined eukaryotic kinase groups) distantly related to the MAPKK proteins (mitogen-activated protein kinase) [97]. Disrupting *Pf*PK7 showed that it was not essential for the asexual blood-stage and was linked to a reduced growth rate during the asexual blood-stage and a lower number of oocysts in the mosquito midgut [98]. *Pf*PK9 is another orphan kinase, essential for the blood-stage [17], with currently only one known phosphorylation target: the E2 ubiquitin-conjugating enzyme 13 (*Pf*UBC13) [99]. The human homolog, *Hs*UBC13, is involved in DNA repair and immune responses [100].

#### 4.14.1. Molecules Targeting *Plasmodium falciparum* Protein Kinase 5 (*Pf*PK5)

**Figure 21:**
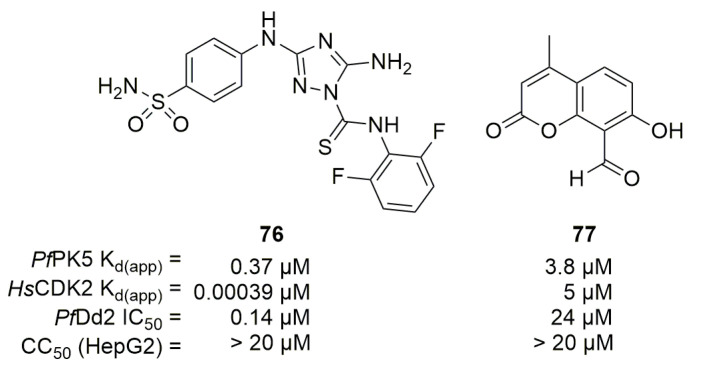
Inhibitors of *Pf*PK5 discovered by Eubanks et al. [95].

Compound **76** is a known *Hs*CDK2 and *Pf*PK5 inhibitor, but is 1000 times more potent toward the human protein (Figure 21). Eubanks et al. synthesized analogs of **76** to try to obtain selective compounds against *Pf*PK5 but their modifications failed [95]. Since *Pf*PK5 crystal structure was available, authors also realized an in-silico screening of 35,000 compounds against *Pf*PK5 followed by an in vitro high throughput binding screening. A chromen-2-one-based compound was found interesting and analogs of this compound were purchased and tested. Compound **77** was the most interesting analog, with a K_d(app)_ of 3.8 µM and 5 µM against *Pf*PK5 and *Hs*CDK2 respectively but lacked activity against *Pf*Dd2 in vitro (Figure 21).

#### 4.14.2. Molecules Targeting *Plasmodium falciparum* Protein Kinase 6 (*Pf*PK6)

**Figure 22:**
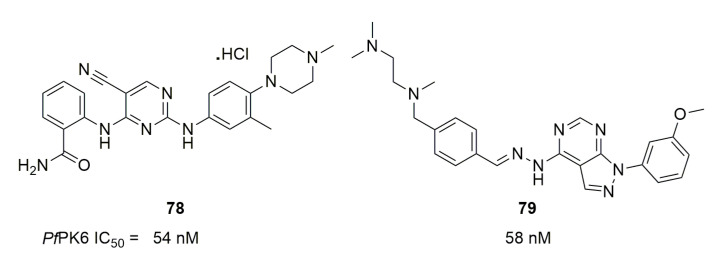
Molecules inhibiting *Pf*PK6 discovered by Crowther et al. [33].

*Pf*PK6 inhibitors have only been identified as hit compounds in the screening by Crowther et al. Figure 22 displays two compounds (**78** and **79**) having an IC_50_ against *Pf*PK6 of around 60 nM but lacked selectivity toward *Pf*PK6.

#### 4.14.3. Molecules Targeting *Plasmodium falciparum* Protein Kinase 7 (*Pf*PK7)

**Figure 23:**
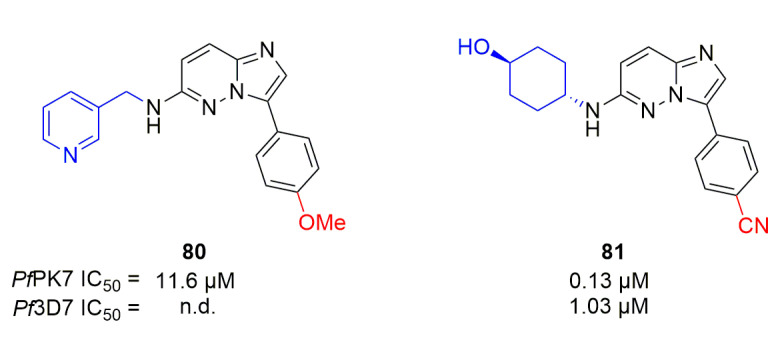
Imidazopyridazine inhibitors of *Pf*PK7.

Starting from hit compound **80** identified by a high-throughput screening campaign, Bouloc et al. synthesized a series of 3-amino-6-phenyl-imidazopyridazines (Figure 23) [101]. Thirty-five analogs were synthesized with modulations targeting the substitution of the amine at position 3 and the nature of the *para*-substituent on the phenyl at position 6. Compound **81** was the best compound of this series, with an IC_50_ of 0.13 µM and 1.09 µM against *Pf*PK7 and *Pf*3D7 respectively.

**Figure 24:**
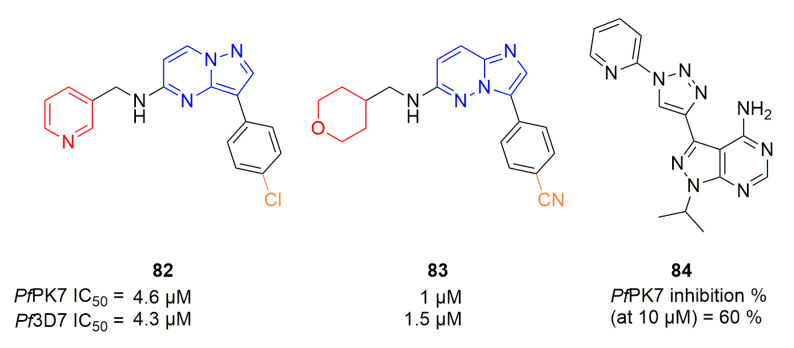
Hits inhibiting *Pf*PK7 discovered by Merckx et al. and Klein et al. [102,103].

Merckx et al. realized a screening of compounds against *Pf*PK7, where two molecules (**82** and **83**, Figure 24) with an imidazopyridazine or pyrazolopyrimidine core displayed moderate activity on both kinase and *Plasmodium* inhibition assays [102]. Klein et al. tested their series of pyrazolopyrimidines against *Pf*PK7 [103]. Compound **84** was shown to inhibit *Pf*PK7 at 10 µM by approximately 60% (Figure 24).

#### 4.14.4. Molecules Targeting *Plasmodium falciparum* Protein Kinase 9 (*Pf*PK9)

*Pf*PK9 inhibitors have only been explored by Raphemot et al. [100]. Starting from a screening of 3,200 molecules, authors discovered that taketinib (**85**), an inhibitor of *Hs*TAK1, was an inhibitor of *Pf*PK9 (Table 11). **85** used at its EC_50_ on *P. berghei* infected HuH7 cells (7.3 µM) was able to increase the size of intracellular parasites but not their numbers, suggesting a deregulation of growth pathways by inhibition of *Pb*PK9. Analogs of taketinib were synthesized to obtain selective compounds against *Pf*PK9: analog **86** displayed a decreased affinity toward *Pf*PK9 at 4.1 µM but was unable to interact with *Hs*TAK1 (Table 11).

### 4.15. Molecules Targeting cGMP Cyclin-Dependent Protein Kinase (PfPKG)

*Pf*PKG is a serine/threonine kinase involved in mechanisms at all the stages of parasite life: parasite motility, hepatocyte invasion, asexual blood-stage development, and gametocytogenesis [104,105,106,107,108]. Compared to *Hs*PKG, *Pf*PKG possesses a smaller gatekeeper (the amino acid at the entrance of the catalytic domain), making it easier to develop molecules selective toward *Pf*PKG. Inhibition of *Pf*PKG led to multistage activity [109,110] with inhibition of:

- sporozoite invasion during the liver-stage,
- development of asexual blood-stage,
- development of gametocytes,
- exflagellation in the mosquito midgut.

Two different scaffolds were explored for *Pf*PKG inhibitors: imidazopyridines and trisubstituted five-membered aromatic cycles which could be viewed as ring-simplified analogs of the first series.

#### 4.15.1. Imidazopyridines Targeting *Pf*PKG

Starting from compound **87** (Table 12), designed originally as a PKG inhibitor against *Eimeria tenella* in chickens [111], Baker et al. realized a SAR study and synthesized nine analogs [110]. This work led to compound **88** with an IC_50_ against *Pf*PKG reduced from 3.1 to 0.16 nM and IC_50_ against *Pf*3D7 strain reduced from 395 to 2.1 nM. **88** was also able to reduce blood-stage growth of *P. berghei* in a mouse model during a four-day Peter’s test by around 60% (25 mg/kg/twice per day *per os*) [27]. Additional work to better understand the properties of the imidazopyridine series was carried out by Large et al., leading to compound **89** [112] which showed lower potency on *P. falciparum* than **87** but better selectivity and metabolic stability.

**Figure 25:**
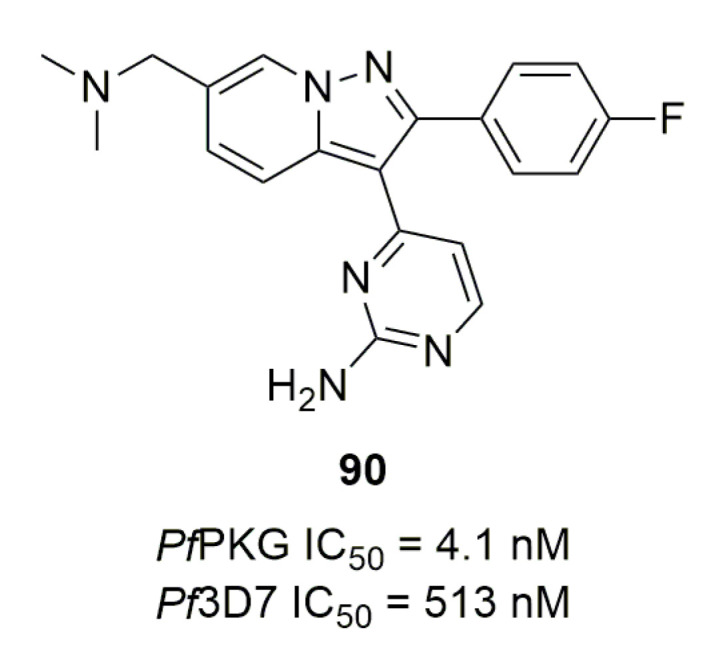
Pyrazolopyridine **90** as inhibitor of *Pf*PKG.

Another study by Large et al. involved modifying the imidazopyridine core using a scaffold hopping strategy [113]. Compound **90**, a pyrazolopyridine analog of **87**, displayed activities on both *Pf*PKG and *Pf*3D7 similar to **87** but with lower metabolic stability (22% of compound remaining after 30 min incubation with mouse liver microsome) due to a more lipophilic molecule (Figure 25).

#### 4.15.2. Trisubstitued Five-Membered Aromatic Cycles Targeting *Pf*PKG

**Figure 26:**
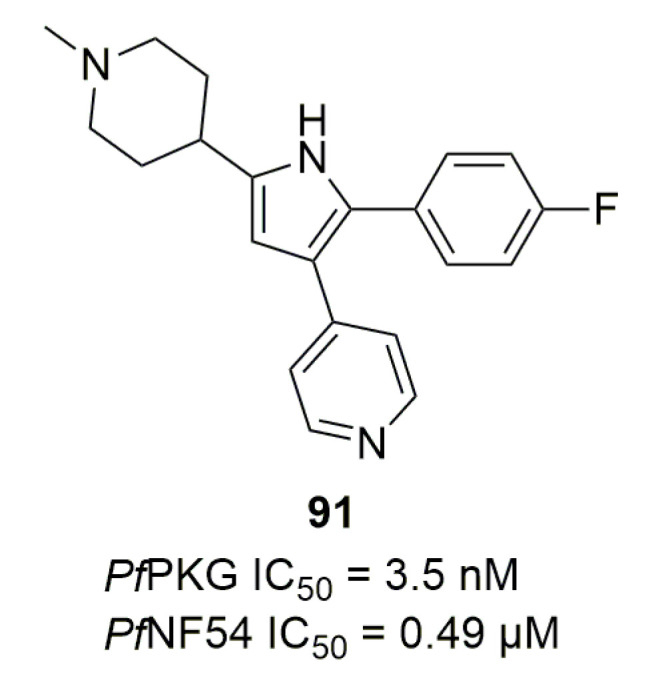
Trisubstituted pyrrole as inhibitor of *Pf*PKG.

Work on trisubstituted five-membered aromatic cycles started with Diaz et al., who tested compound **91** on *P. falciparum* (Figure 26) [114]. **91** was designed initially as an anticoccidial compound targeting *Eimeria tenella* PKG [115]. **91** displayed nanomolar activity against multiple recombinant *Pf*PKG and an IC_50_ of 0.49 µM against *P. falciparum* NF54 strain. Activity in vivo on a mouse model infected by *P. berghei* was also assessed. At 50 mg/kg/7 days by intraperitoneal injections, **91** was able to delay the death of all the mouse groups by 12 days.

**Figure 27:**
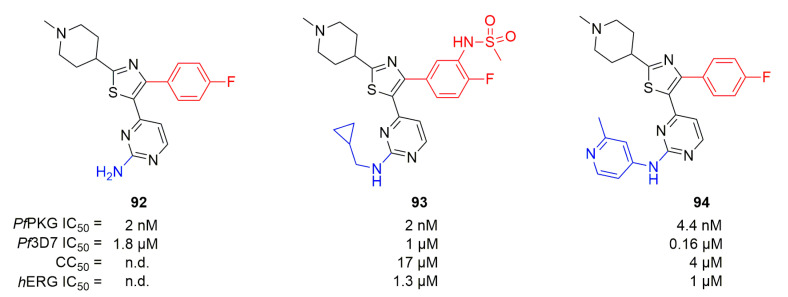
Trisubstituted thiazoles as inhibitors of *Pf*PKG.

Tsagris et al. decided to change the pyrrole core in **91** into a thiazole while keeping key substituents from both compounds **87** and **91** [116]. The authors started from compound **92** which displayed nanomolar inhibition of *Pf*PKG but lacked good potency in vitro against *Pf*3D7 (Figure 27). 15 analogs were synthesized, with modifications targeting all three substituents, leading to compound **93**. Using the same substituents as **87** led to improved potency but **93** displayed an *h*ERG IC_50_ of 1.3 µM (Figure 27). **93** was assessed against human kinases and inhibited all the tested kinases below 70% at 100 nM. Starting from **93**, Matralis et al. continued pharmacomodulations, mainly on the *N*-substituent of aminopyrimidine and the replacement of *N*-methypiperidine [117]. Multiple compounds showed improved potency, including compound **94** (Figure 27). **94** was able to kill parasites rapidly, like artemisinin. Thus, the authors decided to assess **94** in a binding assay against other plasmodial kinases and found that it was also able to bind *Pf*CLK2. This dual activity was linked to the fast-killing profile, since compounds only targeting PfPKG possessed a slow killing profile. Moreover, **94** also showed an affinity toward *Pf*CDPK1 and *Pf*CDPK4.

**Figure 28:**
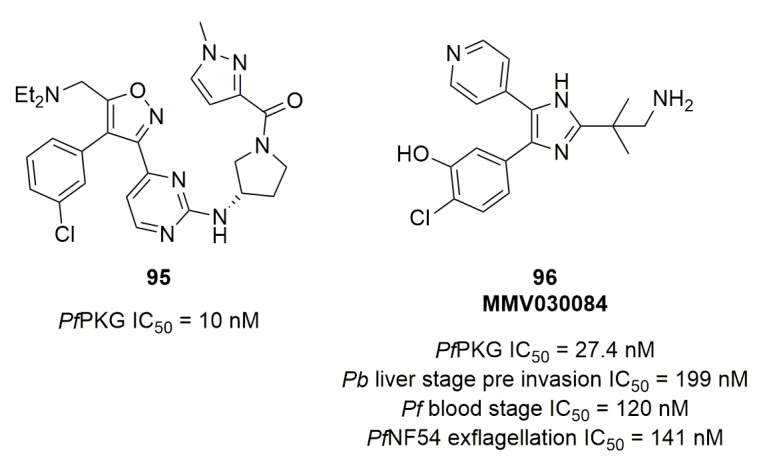
Trisubstituted isoxazole and imidazole as inhibitors of *Pf*PKG.

From three isoxazole-based hits on *Pf*PKG of their own compounds’ library, Ul Mahmood et al. explored the three positions on the isoxazole cycle while keeping key features like aminopyrimidine from previously described *Pf*PKG inhibitors [118]. 29 analogs were synthesized and tested on *Pf*PKG: six of them displayed an IC_50_ below 50 nM, including compound **95**, the most potent one (Figure 28). Finally, MMV030084 (compound **96**, Figure 28), a trisubstituted imidazole, was described by Vanaershot et al. as a multistage active compound (no gametocytocidal effect) targeting *Pf*PKG [109]. Using knockout studies, *Pf*PKG was confirmed to be the main target of **96**.

### 4.16. Molecules Targeting Plasmodium falciparum Thymidylate Kinase (PfTMK)

*Pf*TMK is the enzyme catalyzing the phosphorylation of thymidine monophosphate to thymidine diphosphate. *Pf*TMK is involved in the de novo synthesis of purine bases and is the only way for the parasite to create these bases [119]. This pathway also includes major drug targets such as dihydrofolate reductase (*Pf*DHFR, targeted by sulfadoxine) and more recently, dihydroorotate dehydrogenase (*Pf*DHODH, targeted by DSM265 [120]). *Pf*TMK is also able to catalyze the phosphorylation of deoxyguanosine monophosphate into deoxyguanosine diphosphate.

**Figure 29:**
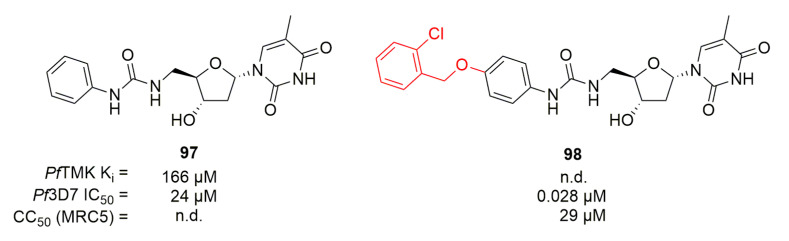
α-thymidine urea analogs as inhibitors of PfTMK.

Research on *Pf*TMK inhibitors has been centered on thymidine analogs. Cui et al. synthesized a large number of thymidine analogs bearing a urea side chain [121]. Compound **97** was used as a starting point for pharmacomodulations which led to compound **98** (Figure 29). Modulations showed several structural features important for potency: α-anomers were more potent than β, thiourea decreased potency whereas hydrophobic *para*-substituent on the phenyl urea increased potency. **98** displayed nanomolar activity on *Pf*3D7 but with low microsomal stability. Surprisingly, no inhibitory assays were performed on the most potent compounds of the series, meaning no clear conclusion can be drawn on the target of these compounds.

**Figure 30:**
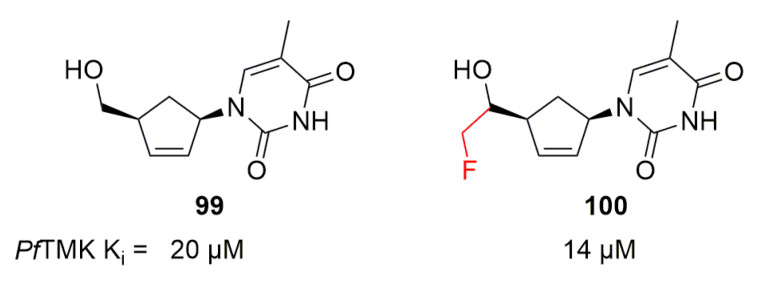
Cyclopentene analogs of thymidine as inhibitors of PfTMK.

Simpler structures were explored by Kato et al., who replaced the tetrahydrofuran by a cyclopentene [122]. This led to compound **99**, showing a weak activity with a K_i_ of 20 µM on *Pf*TMK (Figure 30). **99** was then used by Noguchi et al. as a starting point for pharmacomodulations on the side chain of the cyclopentene core [123]. Compound **100** with a fluoroethanol substituent displayed better potency than **99**, with a K_i_ of 14 µM (Figure 30).

## 5. Conclusions

**Figure 31:**
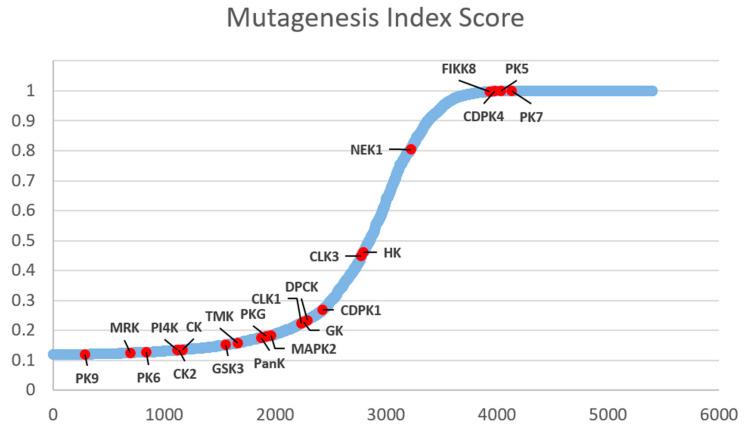
Mutagenesis Index Score graph with protein kinases described in the review highlighted. All *P. falciparum* genes were ordered from the lowest to the highest score and then attributed an arbitrary rank number to obtain this curve. Data from Zhang et al. [124].

Plasmodial kinases appear to be promising targets for new drug therapies against malaria. In the best cases, their inhibition can impact every stage of the parasitic cycle. This is due to the involvement of kinases in most of the cellular mechanisms. Zhang et al. created a mutagenesis index score (MIS) for each gene of the *P. falciparum* asexual blood-stage, illustrating the essentialness of each of these genes (Figure 31) [124]: only five of the kinases described in this review are considered dispensable (MIS close to 1) but two of them (*Pf*CDPK4 and *Pf*PK7) are known to be key proteins during exflagellation.

MMV390048 (**66**), targeting *Pf*PI4K is currently the only plasmodial kinase inhibitor in clinical trial. However, this could quickly change as the way is opened to other *Pf*PI4K inhibitors. This makes *Pf*PI4K inhibitors the most advanced plasmodial kinase inhibitors in the drug development process (Table 13). *Pf*CDPK4 inhibitors are getting close to a pre-clinical stage while *Pf*CDPK1, *Pf*CLK3, *Pf*GSK3 and *Pf*PKG inhibitors are promising compounds awaiting further studies. This represents only six out of the 22 kinases described in this review. Many are still in a hit discovery stage, sometimes without progress for more than ten years.

The development of plasmodial kinase inhibitors is slowed by a lack of X-ray structural data, challenges regarding selectivity against human kinases, and loss of potency going from protein to parasite activity. Moreover, new antiplasmodial hit molecules without a defined target usually have their mechanism of action explored based on commercial antiplasmodial drugs. If these compounds possess multistage activity, they should be tested on plasmodial kinases.

There is still plenty of room for new kinase inhibitors: only 22 out of 86 (or 99) estimated plasmodial kinases were found to be targeted by the compounds in this review. In addition to plasmodial kinases inhibitors, other new promising drugs in development targeting plasmodial proteins such as *Pf*DHODH or *Pf*ATP4 will be important for the creation of new powerful drug combinations to reduce numbers of malaria cases and deaths in years to come.

## Figures and Tables

**Table 1:** Inclusion and exclusion criteria used.

Parameter	Inclusion	Exclusion
Compound’s target	A plasmodial kinase clearly defined as target	Target is not a plasmodial kinase or target is undefined
Study type	Medicinal chemistry studies Biology studies listing properties of one or more chemical compounds	In silico studies without biological tests Natural extract studies without active compounds determined Biological studies on protein pathways/phenotypic changes using only genes disruption or polypeptides

**Table 2:**
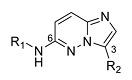
Imidazopyridazine inhibitors of *Pf*CDPK1.

Compound	R_1_	R_2_	*Pf*CDPK1 IC_50_ (nM)	*Pf*3D7 IC_50_ (nM)	In Vivo Parasitemia Reduction ^1^
**5**	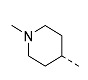	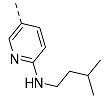	13	400	46
**6**	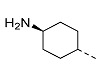	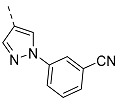	70	103	-
**7**	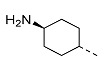	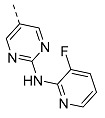	<10	12	4%
**8**	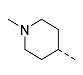	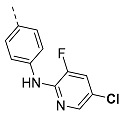	12	80	51%

^1^ Four-day Peter’s test [27] at an oral dose of 50 mg/kg/day.

**Table 3:** ADMET parameters of compound **13** and **14** [37].

	In Vitro		In Vivo		
Compound	Solubility (µM) in Buffer pH 6.5	t_1/2_ (min) Human Liver Microsome	AUC ^1^ (µM·min) at 10 mg/kg per os	AUC ^1^ (µM·min) at 10 mg/kg Intraperitoneal	t_1/2_ (h) at 100 mg/kg per os
**13**	47	-	57	317	-
**14**	82	>60	430	863	13.5

^1^ AUC = Area Under Curve.

**Table 4:** Bispyridinium bromide salt and bisquinolinium bromide salt inhibitors of *Pf*CK.

Compound	*Pf*CK_Cho_ IC_50_ (µM)	*Pf*3D7 IC_50_ (nM)	*Hs*CKα1 IC_50_
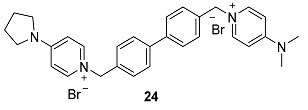	276	3	-
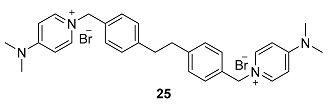	103	3	-
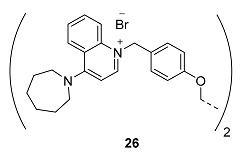	2.4	142	1.7

**Table 5:** Summary of in vitro properties of *Pf*CLK inhibitors from Kern et al. [53].

Compound	*Pf*3D7 IC_50_ (µM)	EIA ^1^ IC_50_ (µM)	% Inhibition (at *Pf*3D7 IC_50_ Concentration)			
			*Pf*CLK1	*Pf*CLK2	*Pf*CLK3	*Pf*CLK4
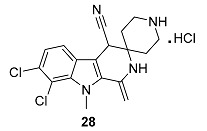	4.4	13.8	73%	45.6%	35.1%	71.6%
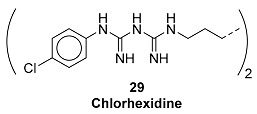	0.6	19.8	68.4%	76.4%	74.2%	76.1%

^1^ Exflagellation Inhibitory Assay.

**Table 6:** Pantothenate and pantothenate analog inhibitors of *Pf*PanK.

Compound	*P. falciparum* IC_50_ (µM) ^1,2^	Pantothenate Phosphorylation Inhibition %	*Pf*PanK IC_50_
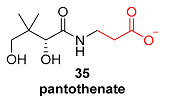	-	-	-
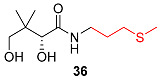	76	>85% (at 1 mM)	-
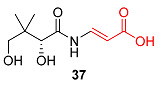	36	-	14 µM
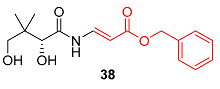	13	>60% (at 10 µM)	140 nM
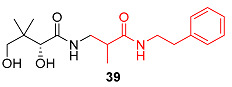	0.03	-	-

^1^*Pf3D7* strain except for **39**, where the value shown is on *Pf*W2 strain. ^2^ Pantothenate concentration in growth medium was 1 µM except for **39**, where it was 1.14 µM.

**Table 7:**
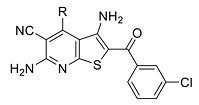
Thieno[2,3-b]pyridine inhibitors of *Pf*GSK3.

Compound	R	% Inhibition *Pf*NF54LUC at 3 µM	*Pf*GSK3 IC_50_ (µM)	*Hs*GSK3 IC_50_ (µM)	*Pf*NF54LUC IC_50_ (µM)	CC_50_ (HEK293, µM)
**48**	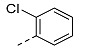	n.d. ^1^	0.24	9.08	5.5	35.2
**49**	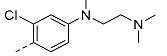	99.9	0.72	40.2	1.2	5.56
(+)-**50**	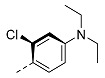	73.0	n.d.	n.d.	1.1	n.d.
(−)-**50**	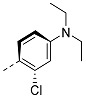	−24.4	n.d.	n.d.	n.d.	n.d.

^1^ n.d. = not determined

**Table 8:**
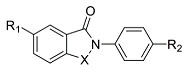
Isobenzothiazolinones and ebselen as inhibitors of *Pf*HK.

Compound	X	R_1_	R_2_	*Pf*HK IC_50_ (nM)	*Pf*3D7 EC_50_ (µM)
**51**	S	F	Cl	160	0.62
**52**	S	H	Cl	270	1.1
**53**	S	H	H	230	5.2
**54**	S	H	H	10	6.8

**Table 9:**
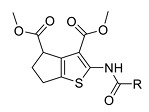
Dimethyl 2-amido-cyclopenta[*b*]thiophene-3,4-dicarboxylates as inhibitors of *Pf*HK.

Compound	R	*Pf*HK IC_50_ (µM)
**55**	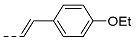	1.09
**56**	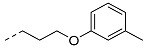	1.94

**Table 10:** Three of the inhibitors of *Pf*PI4K described by McNamara et al. [85].

Compound	*P. yoelii* liver stage IC_50_ (nM)	*P. falciparum* Asexual Blood-Stage IC_50_ (nM)	*Pf*NF54 Gametocyte III IC_50_ (nM)
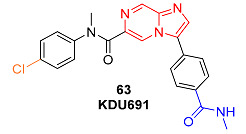	36	29.9	220
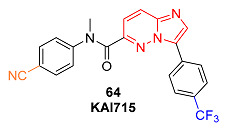	9	3.9	n.d. ^1^
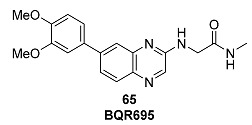	n.d.	71	n.d.

^1^ n.d. = not determined

**Table 11:**
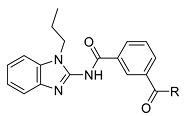
Taketinib and its analog as inhibitors of *Pf*PK9.

Compound	R	K_d(app)_ *Pf*PK9 (µM)	HuH7 *P. berghei* Infected Cell Load EC_50_ (µM)
**85 (taketinib)**	NH_2_	0.46	7.3
**86**	OH	4.1	43

**Table 12:**
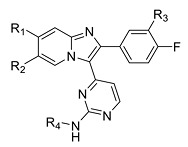
Imidazopyridines as inhibitors of *Pf*PKG.

Compound	R_1_	R_2_	R_3_	R_4_	*Pf*PKG IC_50_ (nM)	*Pf*3D7 IC_50_ (nM)	% MLM Remaining ^1^
**87**	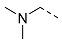	H	H	H	3.1	395	52
**88**	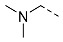	Me	-NHSO_2_Me	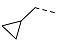	0.16	2.1	-
**89**	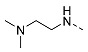	H	H	Me	2.8	525	93

^1^ % remaining after 30 min incubation with mouse liver microsomes.

**Table 13:** Plasmodial kinases targeted by inhibiting compounds described in this review.

Protein	Role of Protein—Consequence(s) of Inhibition ^1^	Development Stage of the Most Advanced Compound(s)	Studies Reviewed
CDPK1	Egress and invasion processes regulator—Inhibition of asexual blood-stage growth and gametogenesis, reduced mosquito infection	Hit to lead	[23,24,28,29,30,31,32,33]
CDPK4	Exflagellation regulator—Reduced mosquito infection	Lead optimization	[33,36,37,38,39]
CK	Transformation of choline into phosphocholine	Hit discovery—Target validation needed	[41,45,46,47]
CK2	Serine/threonine kinase, possibly phosphorylating many proteins during asexual blood-stage	Hit discovery—In vitro activity to be determined	[50]
CLK1	Phosphorylation of spliceosomes proteins—Inhibition of asexual and sexual blood-stage growth	Hit discovery—In vitro activity to be determined	[53,55]
CLK3	Phosphorylation of spliceosomes proteins—All-stage activity	Hit to lead	[53,54,56]
PanK	Transformation of pantothenate into 4′-phosphopantothenate	Hit discovery	[58,59,60]
DPCK	Transformation of dephospho-coenzyme A into coenzyme A	Hit discovery	[61]
FKk8	Not defined	Hit discovery	[64,65]
GK	Transformation of (deoxy) guanosine-monophosphate into (deoxy) guanosine-diphosphate	Hit discovery—Target validation needed	[46]
GSK3	Not defined	Hit to lead	[70,71]
HK	Transformation of glucose into glucose-6-phosphate	Hit discovery	[74,75]
MAP2	Involved in many key cellular processes—Inhibition of asexual blood-stage and exflagellation	Hit discovery—Target validation needed	[77]
MRK	Regulation of gene expression and DNA replication	Hit discovery	[79,80,81]
NEK1	Regulation of cell division processes	Hit discovery	[83,84]
PI4K	Transformation of phosphatidyl-inositol into phosphatidyl-inositol 4 phosphate—All-stage activity	Phase II clinical trial	[85,87,89,90,91,92]
PK5	Cyclin-dependent like kinase, possibly regulating cell division	Hit discovery	[95]
PK6	Cyclin-dependent like kinase, role not defined	Hit discovery - In vitro activity to be determined	[33]
PK7	Orphan kinase, role not defined—Inhibition of asexual blood-stage and reduced mosquito infection	Hit to lead	[101,102,103]
PK9	Orphan kinase, role unclear, able to phosphorylate *Pf*UBC13—Deregulation of asexual hepatic stage growth	Hit discovery	[100]
PKG	Serine/threonine kinase—All-stage activity	Hit to lead	[109,110,112,113,114,116,117,118]
TMK	Transformation of thymidine monophosphate into thymidine diphosphate	Hit to lead	[121,122,123]

^1^ When not specified, each of these proteins is assumed to at least inhibit asexual blood-stage growth.

## References

[1] (2019). World Health Organization World Malaria Report 2019.

[2] World Health Organization, Global Malaria Programme (2015). Global Technical Strategy for Malaria, 2016–2030.

[3] Ranson H., Lissenden N. (2016). Insecticide Resistance in African *Anopheles* Mosquitoes: A Worsening Situation that Needs Urgent Action to Maintain Malaria Control. Trends Parasitol..

[4] Birnbaum J., Scharf S., Schmidt S., Jonscher E., Hoeijmakers W.A.M., Flemming S., Toenhake C.G., Schmitt M., Sabitzki R., Bergmann B. (2020). A Kelch13-defined endocytosis pathway mediates artemisinin resistance in malaria parasites. Science.

[5] Ouji M., Augereau J.-M., Paloque L., Benoit-Vical F. (2018). *Plasmodium falciparum* resistance to artemisinin-based combination therapies: A sword of Damocles in the path toward malaria elimination. Parasite.

[6] Phyo A.P., Win K.K., Thu A.M., Swe L.L., Htike H., Beau C., Sriprawat K., Winterberg M., Proux S., Imwong M. (2018). Poor response to artesunate treatment in two patients with severe malaria on the Thai–Myanmar border. Malaria J..

[7] Uwimana A., Legrand E., Stokes B.H., Ndikumana J.-L.M., Warsame M., Umulisa N., Ngamije D., Munyaneza T., Mazarati J.-B., Munguti K. (2020). Emergence and clonal expansion of in vitro artemisinin-resistant *Plasmodium falciparum* kelch13 R561H mutant parasites in Rwanda. Nat. Med..

[8] Sinka M.E., Pironon S., Massey N.C., Longbottom J., Hemingway J., Moyes C.L., Willis K.J. (2020). A new malaria vector in Africa: Predicting the expansion range of *Anopheles stephensi* and identifying the urban populations at risk. Proc. Natl. Acad. Sci. USA.

[9] Burrows J.N., Duparc S., Gutteridge W.E., Hooft van Huijsduijnen R., Kaszubska W., Macintyre F., Mazzuri S., Möhrle J.J., Wells T.N.C. (2017). New developments in anti-malarial target candidate and product profiles. Malaria J..

[10] Manning G. (2002). The Protein Kinase Complement of the Human Genome. Science.

[11] Cohen P. (2002). Protein kinases—The major drug targets of the twenty-first century?. Nat. Rev. Drug. Discov..

[12] Roskoski R. (2019). Properties of FDA-approved small molecule protein kinase inhibitors. Pharmacol. Res..

[13] Carles F., Bourg S., Meyer C., Bonnet P. (2018). PKIDB: A Curated, Annotated and Updated Database of Protein Kinase Inhibitors in Clinical Trials. Molecules.

[14] Ward P., Equinet L., Packer J., Doerig C. (2004). Protein kinases of the human malaria parasite *Plasmodium falciparum*: The kinome of a divergent eukaryote. BMC Genom..

[15] Anamika, Srinivasan N., Krupa A. (2004). A genomic perspective of protein kinases in *Plasmodium falciparum*. Proteins.

[16] Lucet I.S., Tobin A., Drewry D., Wilks A.F., Doerig C. (2012). Plasmodium kinases as targets for new-generation antimalarials. Future Med. Chem..

[17] Solyakov L., Halbert J., Alam M.M., Semblat J.-P., Dorin-Semblat D., Reininger L., Bottrill A.R., Mistry S., Abdi A., Fennell C. (2011). Global kinomic and phospho-proteomic analyses of the human malaria parasite *Plasmodium falciparum*. Nat. Commun..

[18] Tewari R., Straschil U., Bateman A., Böhme U., Cherevach I., Gong P., Pain A., Billker O. (2010). The Systematic Functional Analysis of *Plasmodium* Protein Kinases Identifies Essential Regulators of Mosquito Transmission. Cell Host Microbe.

[19] Moher D., Liberati A., Tetzlaff J., Altman D.G., Group T.P. (2009). Preferred Reporting Items for Systematic Reviews and Meta-Analyses: The PRISMA Statement. PLoS Med..

[20] Harper J.F., Harmon A. (2005). Plants, symbiosis and parasites: A calcium signalling connection. Nat. Rev. Mol. Cell. Bio..

[21] Billker O., Lourido S., Sibley L.D. (2009). Calcium-Dependent Signaling and Kinases in Apicomplexan Parasites. Cell Host Microbe.

[22] Zhao Y., Kappes B., Franklin R. (1993). Gene Structure and Expression of an Unusual Protein Kinase from *Plasmodium falciparum* Homologous at Its Carboxyl Terminus with the EF Hand Calcium-binding Proteins. J. Biol. Chem..

[23] Green J.L., Rees-Channer R.R., Howell S.A., Martin S.R., Knuepfer E., Taylor H.M., Grainger M., Holder A.A. (2008). The motor complex of *Plasmodium falciparum*: Phosphorylation by a calcium-dependent protein kinase. J. Biol. Chem..

[24] Kato N., Sakata T., Breton G., Le Roch K.G., Nagle A., Andersen C., Bursulaya B., Henson K., Johnson J., Kumar K.A. (2008). Gene expression signatures and small-molecule compounds link a protein kinase to *Plasmodium falciparum* motility. Nat. Chem. Biol..

[25] Bansal A., Molina-Cruz A., Brzostowski J., Liu P., Luo Y., Gunalan K., Li Y., Ribeiro J.M.C., Miller L.H. (2018). *Pf*CDPK1 is critical for malaria parasite gametogenesis and mosquito infection. Proc. Natl. Acad. Sci. USA.

[26] Kumar S., Kumar M., Ekka R., Dvorin J.D., Paul A.S., Madugundu A.K., Gilberger T., Gowda H., Duraisingh M.T., Keshava Prasad T.S. (2017). *Pf*CDPK1 mediated signaling in erythrocytic stages of *Plasmodium falciparum*. Nat. Commun..

[27] Peters W., Robinson B.L., Zak O., Sande M.A. (1999). Chapter 92—Malaria. Handbook of Animal Models of Infection.

[28] Lemercier G., Fernandez-Montalvan A., Shaw J.P., Kugelstadt D., Bomke J., Domostoj M., Schwarz M.K., Scheer A., Kappes B., Leroy D. (2009). Identification and characterization of novel small molecules as potent inhibitors of the plasmodial calcium-dependent protein kinase 1. Biochemistry.

[29] Ansell K.H., Jones H.M., Whalley D., Hearn A., Taylor D.L., Patin E.C., Chapman T.M., Osborne S.A., Wallace C., Birchall K. (2014). Biochemical and antiparasitic properties of inhibitors of the *Plasmodium falciparum* calcium-dependent protein kinase *Pf*CDPK1. Antimicrob. Agents Chemother..

[30] Chapman T.M., Osborne S.A., Bouloc N., Large J.M., Wallace C., Birchall K., Ansell K.H., Jones H.M., Taylor D., Clough B. (2013). Substituted imidazopyridazines are potent and selective inhibitors of *Plasmodium falciparum* calcium-dependent protein kinase 1 (*Pf*CDPK1). Bioorg. Med. Chem. Lett..

[31] Large J.M., Osborne S.A., Smiljanic-Hurley E., Ansell K.H., Jones H.M., Taylor D.L., Clough B., Green J.L., Holder A.A. (2013). Imidazopyridazines as potent inhibitors of *Plasmodium falciparum* calcium-dependent protein kinase 1 (*Pf*CDPK1): Preparation and evaluation of pyrazole linked analogues. Bioorg. Med. Chem. Lett..

[32] Chapman T.M., Osborne S.A., Wallace C., Birchall K., Bouloc N., Jones H.M., Ansell K.H., Taylor D.L., Clough B., Green J.L. (2014). Optimization of an imidazopyridazine series of inhibitors of *Plasmodium falciparum* calcium-dependent protein kinase 1 (*Pf*CDPK1). J. Med. Chem..

[33] Crowther G.J., Hillesland H.K., Keyloun K.R., Reid M.C., Lafuente-Monasterio M.J., Ghidelli-Disse S., Leonard S.E., He P., Jones J.C., Krahn M.M. (2016). Biochemical Screening of Five Protein Kinases from *Plasmodium falciparum* against 14,000 Cell-Active Compounds. PLoS ONE.

[34] Billker O., Dechamps S., Tewari R., Wenig G., Franke-Fayard B., Brinkmann V. (2004). Calcium and a Calcium-Dependent Protein Kinase Regulate Gamete Formation and Mosquito Transmission in a Malaria Parasite. Cell.

[35] Kato K., Sudo A., Kobayashi K., Sugi T., Tohya Y., Akashi H. (2009). Characterization of *Plasmodium falciparum* calcium-dependent protein kinase 4. Parasitol. Int..

[36] Ojo K.K., Pfander C., Mueller N.R., Burstroem C., Larson E.T., Bryan C.M., Fox A.M.W., Reid M.C., Johnson S.M., Murphy R.C. (2012). Transmission of malaria to mosquitoes blocked by bumped kinase inhibitors. J. Clin. Invest..

[37] Ojo K.K., Eastman R.T., Vidadala R., Zhang Z., Rivas K.L., Choi R., Lutz J.D., Reid M.C., Fox A.M.W., Hulverson M.A. (2014). A specific inhibitor of *Pf*CDPK4 blocks malaria transmission: Chemical-genetic validation. J. Infect. Dis..

[38] Vidadala R.S.R., Ojo K.K., Johnson S.M., Zhang Z., Leonard S.E., Mitra A., Choi R., Reid M.C., Keyloun K.R., Fox A.M.W. (2014). Development of potent and selective *Plasmodium falciparum* calcium-dependent protein kinase 4 (*Pf*CDPK4) inhibitors that block the transmission of malaria to mosquitoes. Eur. J. Med. Chem..

[39] Huang W., Hulverson M.A., Zhang Z., Choi R., Hart K.J., Kennedy M., Vidadala R.S.R., Maly D.J., Van Voorhis W.C., Lindner S.E. (2016). Aminopyrazole-4-carboxamide analogues are selective inhibitors of *Plasmodium falciparum* microgametocyte exflagellation and potential malaria transmission blocking agents. Bioorg. Med. Chem. Lett..

[40] Huang W., Hulverson M.A., Choi R., Arnold S.L.M., Zhang Z., McCloskey M.C., Whitman G.R., Hackman R.C., Rivas K.L., Barrett L.K. (2019). Development of 5-Aminopyrazole-4-carboxamide-based Bumped-Kinase Inhibitors for Cryptosporidiosis Therapy. J. Med. Chem..

[41] Serran-Aguilera L., Denton H., Rubio-Ruiz B., Lopez-Gutierrez B., Entrena A., Izquierdo L., Smith T.K., Conejo-Garcia A., Hurtado-Guerrero R. (2016). *Plasmodium falciparum* Choline Kinase Inhibition Leads to a Major Decrease in Phosphatidylethanolamine Causing Parasite Death. Sci. Rep..

[42] Ancelin M.L., Calas M., Bompart J., Cordina G., Martin D., Ben Bari M., Jei T., Druilhe P., Vial H.J. (1998). Antimalarial Activity of 77 Phospholipid Polar Head Analogs: Close Correlation Between Inhibition of Phospholipid Metabolism and In Vitro *Plasmodium Falciparum* Growth. Blood.

[43] Ancelin M.L., Calas M., Vidal-Sailhan V., Herbuté S., Ringwald P., Vial H.J. (2003). Potent Inhibitors of *Plasmodium* Phospholipid Metabolism with a Broad Spectrum of In Vitro Antimalarial Activities. Antimicrob. Agents Chemoter..

[44] Serran-Aguilera L., Nuti R., Lopez-Cara L.C., Rios-Marco P., Carrasco M.P., Marco C., Entrena A., Macchiarulo A., Hurtado-Guerrero R. (2014). Choline Kinase Active Site Provides Features for Designing Versatile Inhibitors. Curr. Top. Med. Chem..

[45] Choubey V., Maity P., Guha M., Kumar S., Srivastava K., Puri S.K., Bandyopadhyay U. (2007). Inhibition of *Plasmodium falciparum* choline kinase by hexadecyltrimethylammonium bromide: A possible antimalarial mechanism. Antimicrob. Agents Chemoter..

[46] Crowther G.J., Napuli A.J., Gilligan J.H., Gagaring K., Borboa R., Francek C., Chen Z., Dagostino E.F., Stockmyer J.B., Wang Y. (2011). Identification of inhibitors for putative malaria drug targets among novel antimalarial compounds. Mol. Biochem. Parasit..

[47] Schiafino-Ortega S., Baglioni E., Perez-Moreno G., Marco P.R., Marco C., Gonzalez-Pacanowska D., Ruiz-Perez L.M., Carrasco-Jimenez M.P., Lopez-Cara L.C. (2018). 1,2-Diphenoxiethane salts as potent antiplasmodial agents. Bioorg. Med. Chem. Lett..

[48] Pease B.N., Huttlin E.L., Jedrychowski M.P., Talevich E., Harmon J., Dillman T., Kannan N., Doerig C., Chakrabarti R., Gygi S.P. (2013). Global Analysis of Protein Expression and Phosphorylation of Three Stages of *Plasmodium falciparum* Intraerythrocytic Development. J. Proteome Res..

[49] Holland Z., Prudent R., Reiser J.-B., Cochet C., Doerig C. (2009). Functional Analysis of Protein Kinase CK2 of the Human Malaria Parasite *Plasmodium falciparum*. Eukaryot. Cell.

[50] Ruiz-Carrillo D., Lin J., El Sahili A., Wei M., Sze S.K., Cheung P.C.F., Doerig C., Lescar J. (2018). The protein kinase CK2 catalytic domain from Plasmodium falciparum: Crystal structure, tyrosine kinase activity and inhibition. Sci. Rep..

[51] Testing the Safety and Tolerability of CX-4945 in Patients with Recurrent Medulloblastoma Who May or May Not Have Surgery—ClinicalTrials.gov. https://clinicaltrials.gov/ct2/show/NCT03904862.

[52] Talevich E., Mirza A., Kannan N. (2011). Structural and evolutionary divergence of eukaryotic protein kinases in *Apicomplexa*. BMC Evol. Biol..

[53] Kern S., Agarwal S., Huber K., Gehring A.P., Strödke B., Wirth C.C., Brügl T., Abodo L.O., Dandekar T., Doerig C. (2014). Inhibition of the SR protein-phosphorylating CLK kinases of *Plasmodium falciparum* impairs blood stage replication and malaria transmission. PLoS ONE.

[54] Alam M.M., Sanchez-Azqueta A., Janha O., Flannery E.L., Mahindra A., Mapesa K., Char A.B., Sriranganadane D., Brancucci N.M.B., Antonova-Koch Y. (2019). Validation of the protein kinase PfCLK3 as a multistage cross-species malarial drug target. Science.

[55] Bendjeddou L.Z., Loaëc N., Villiers B., Prina E., Späth G.F., Galons H., Meijer L., Oumata N. (2017). Exploration of the imidazo[1,2-*b*]pyridazine scaffold as a protein kinase inhibitor. Eur. J. Med. Chem..

[56] Mahindra A., Janha O., Mapesa K., Sanchez-Azqueta A., Alam M.M., Amambua-Ngwa A., Nwakanma D.C., Tobin A.B., Jamieson A.G. (2020). Development of Potent *Pf*CLK3 Inhibitors Based on TCMDC-135051 as a New Class of Antimalarials. J. Med. Chem..

[57] Genschel U. (2004). Coenzyme A Biosynthesis: Reconstruction of the Pathway in Archaea and an Evolutionary Scenario Based on Comparative Genomics. Mol. Biol. Evol..

[58] Spry C., Chai C.L.L., Kirk K., Saliba K.J. (2005). A class of pantothenic acid analogs inhibits *Plasmodium falciparum* pantothenate kinase and represses the proliferation of malaria parasites. Antimicrob. Agents Chemother..

[59] Spry C., Sewell A.L., Hering Y., Villa M.V.J., Weber J., Hobson S.J., Harnor S.J., Gul S., Marquez R., Saliba K.J. (2018). Structure-activity analysis of CJ-15,801 analogues that interact with *Plasmodium falciparum* pantothenate kinase and inhibit parasite proliferation. Eur. J. Med. Chem..

[60] Chiu J.E., Thekkiniath J., Choi J.-Y., Perrin B.A., Lawres L., Plummer M., Virji A.Z., Abraham A., Toh J.Y., Zandt M.V. (2017). The antimalarial activity of the pantothenamide α-PanAm is via inhibition of pantothenate phosphorylation. Sci. Rep..

[61] Fletcher S., Avery V.M. (2014). A novel approach for the discovery of chemically diverse anti-malarial compounds targeting the *Plasmodium falciparum* Coenzyme A synthesis pathway. Malaria J..

[62] Brandt G.S., Bailey S. (2013). Dematin, a human erythrocyte cytoskeletal protein, is a substrate for a recombinant FIKK kinase from *Plasmodium falciparum*. Mol. Biochem. Parasit..

[63] Kats L.M., Fernandez K.M., Glenister F.K., Herrmann S., Buckingham D.W., Siddiqui G., Sharma L., Bamert R., Lucet I., Guillotte M. (2014). An exported kinase (FIKK4.2) that mediates virulence-associated changes in *Plasmodium falciparum*-infected red blood cells. Int. J. Parasitol..

[64] Lin B.C., Harris D.R., Kirkman L.M.D., Perez A.M., Qian Y., Schermerhorn J.T., Hong M.Y., Winston D.S., Xu L., Lieber A.M. (2017). The anthraquinone emodin inhibits the non-exported FIKK kinase from *Plasmodium falciparum*. Bioorg. Chem..

[65] Lin B.C., Harris D.R., Kirkman L.M.D., Perez A.M., Qian Y., Schermerhorn J.T., Hong M.Y., Winston D.S., Xu L., Brandt G.S. (2017). FIKK Kinase, a Ser/Thr Kinase Important to Malaria Parasites, Is Inhibited by Tyrosine Kinase Inhibitors. ACS Omega.

[66] Kandeel M., Kitade Y. (2008). Molecular Characterization, Heterologous Expression and Kinetic Analysis of Recombinant *Plasmodium falciparum* Thymidylate Kinase. J. Biochem..

[67] Tarun A.S., Peng X., Dumpit R.F., Ogata Y., Silva-Rivera H., Camargo N., Daly T.M., Bergman L.W., Kappe S.H.I. (2008). A combined transcriptome and proteome survey of malaria parasite liver stages. Proc. Natl. Acad. Sci. USA.

[68] Prinz B., Harvey K.L., Wilcke L., Ruch U., Engelberg K., Biller L., Lucet I., Erkelenz S., Heincke D., Spielmann T. (2016). Hierarchical phosphorylation of apical membrane antigen 1 is required for efficient red blood cell invasion by malaria parasites. Sci. Rep..

[69] Saraswati A.P., Ali Hussaini S.M., Krishna N.H., Babu B.N., Kamal A. (2018). Glycogen synthase kinase-3 and its inhibitors: Potential target for various therapeutic conditions. Eur. J. Med. Chem..

[70] Fugel W., Oberholzer A.E., Gschloessl B., Dzikowski R., Pressburger N., Preu L., Pearl L.H., Baratte B., Ratin M., Okun I. (2013). 3,6-Diamino-4-(2-halophenyl)-2-benzoylthieno[2,3-b]pyridine-5-carbonitriles are selective inhibitors of *Plasmodium falciparum* glycogen synthase kinase-3. J. Med. Chem..

[71] Masch A., Nasereddin A., Alder A., Bird M.J., Schweda S.I., Preu L., Doerig C., Dzikowski R., Gilberger T.W., Kunick C. (2019). Structure-activity relationships in a series of antiplasmodial thieno[2,3-b]pyridines. Malaria J..

[72] Slavic K., Straschil U., Reininger L., Doerig C., Morin C., Tewari R., Krishna S. (2010). Life cycle studies of the hexose transporter of *Plasmodium* species and genetic validation of their essentiality. Mol. Microbiol..

[73] van Schalkwyk D.A., Priebe W., Saliba K.J. (2008). The Inhibitory Effect of 2-Halo Derivatives of d-Glucose on Glycolysis and on the Proliferation of the Human Malaria Parasite *Plasmodium falciparum*. J. Pharmacol. Exp. Ther..

[74] Harris M.T., Walker D.M., Drew M.E., Mitchell W.G., Dao K., Schroeder C.E., Flaherty D.P., Weiner W.S., Golden J.E., Morris J.C. (2013). Interrogating a Hexokinase-Selected Small-Molecule Library for Inhibitors of *Plasmodium falciparum* Hexokinase. Antimicrob. Agents Chemother..

[75] Davis M.I., Patrick S.L., Blanding W.M., Dwivedi V., Suryadi J., Golden J.E., Coussens N.P., Lee O.W., Shen M., Boxer M.B. (2016). Identification of Novel *Plasmodium falciparum* Hexokinase Inhibitors with Antiparasitic Activity. Antimicrob. Agents Chemother..

[76] Hitz E., Balestra A.C., Brochet M., Voss T.S. (2020). PfMAP-2 is essential for male gametogenesis in the malaria parasite *Plasmodium falciparum*. Sci. Rep..

[77] Brumlik M.J., Nkhoma S., Kious M.J., Thompson G.R., Patterson T.F., Siekierka J.J., Anderson T.J.C., Curiel T.J. (2011). Human p38 mitogen-activated protein kinase inhibitor drugs inhibit *Plasmodium falciparum* replication. Exp. Parasitol..

[78] Jirage D., Chen Y., Caridha D., O’Neil M.T., Eyase F., Witola W.H., Mamoun C.B., Waters N.C. (2010). The malarial CDK *Pf*mrk and its effector *Pf*MAT1 phosphorylate DNA replication proteins and co-localize in the nucleus. Mol. Biochem. Parasit..

[79] Woodard C.L., Keenan S.M., Gerena L., Welsh W.J., Geyer J.A., Waters N.C. (2007). Evaluation of broad spectrum protein kinase inhibitors to probe the architecture of the malarial cyclin dependent protein kinase *Pf*mrk. Bioorg. Med. Chem. Lett..

[80] Geyer J.A., Keenan S.M., Woodard C.L., Thompson P.A., Gerena L., Nichols D.A., Gutteridge C.E., Waters N.C. (2009). Selective inhibition of Pfmrk, a *Plasmodium falciparum* CDK, by antimalarial 1,3-diaryl-2-propenones. Bioorg. Med. Chem. Lett..

[81] Caridha D., Kathcart A.K., Jirage D., Waters N.C. (2010). Activity of substituted thiophene sulfonamides against malarial and mammalian cyclin dependent protein kinases. Bioorg. Med. Chem. Lett..

[82] Carvalho T.G., Doerig C., Reininger L. (2013). Nima- and Aurora-related kinases of malaria parasites. Biochim. Biophys. Acta.

[83] Laurent D., Jullian V., Parenty A., Knibiehler M., Dorin D., Schmitt S., Lozach O., Lebouvier N., Frostin M., Alby F. (2006). Antimalarial potential of xestoquinone, a protein kinase inhibitor isolated from a Vanuatu marine sponge *Xestospongia sp*. Bioorg. Med. Chem..

[84] Desoubzdanne D., Marcourt L., Raux R., Chevalley S., Dorin D., Doerig C., Valentin A., Ausseil F., Debitus C. (2008). Alisiaquinones and Alisiaquinol, Dual Inhibitors of *Plasmodium falciparum* Enzyme Targets from a New Caledonian Deep Water Sponge. J. Nat. Prod..

[85] McNamara C.W., Lee M.C.S., Lim C.S., Lim S.H., Roland J., Nagle A., Simon O., Yeung B.K.S., Chatterjee A.K., McCormack S.L. (2013). Targeting *Plasmodium* PI(4)K to eliminate malaria. Nature.

[86] MMV390048 POC in Patients with *P. vivax* and *P. falciparum* Malaria—ClinicalTrials.gov. https://clinicaltrials.gov/ct2/show/NCT02880241.

[87] Paquet T., Le Manach C., Cabrera D.G., Younis Y., Henrich P.P., Abraham T.S., Lee M.C.S., Basak R., Ghidelli-Disse S., Lafuente-Monasterio M.J. (2017). Antimalarial efficacy of MMV390048, an inhibitor of *Plasmodium* phosphatidylinositol. Sci. Transl. Med..

[88] Younis Y., Douelle F., Feng T.-S., Cabrera D.G., Manach C.L., Nchinda A.T., Duffy S., White K.L., Shackleford D.M., Morizzi J. (2012). 3,5-Diaryl-2-aminopyridines as a Novel Class of Orally Active Antimalarials Demonstrating Single Dose Cure in Mice and Clinical Candidate Potential. J. Med. Chem..

[89] Brunschwig C., Lawrence N., Taylor D., Abay E., Njoroge M., Basarab G.S., Le Manach C., Paquet T., Cabrera D.G., Nchinda A.T. (2018). UCT943, a Next-Generation *Plasmodium falciparum* PI4K Inhibitor Preclinical Candidate for the Treatment of Malaria. Antimicrob. Agents Chemother..

[90] Gibhard L., Njoroge M., Paquet T., Brunschwig C., Taylor D., Lawrence N., Abay E., Wittlin S., Wiesner L., Street L.J. (2018). Investigating Sulfoxide-to-Sulfone Conversion as a Prodrug Strategy for a Phosphatidylinositol 4-Kinase Inhibitor in a Humanized Mouse Model of Malaria. Antimicrob. Agents Chemother..

[91] Kandepedu N., Gonzàlez Cabrera D., Eedubilli S., Taylor D., Brunschwig C., Gibhard L., Njoroge M., Lawrence N., Paquet T., Eyermann C.J. (2018). Identification, Characterization, and Optimization of 2,8-Disubstituted-1,5-naphthyridines as Novel *Plasmodium falciparum* Phosphatidylinositol-4-kinase Inhibitors with in Vivo Efficacy in a Humanized Mouse Model of Malaria. J. Med. Chem..

[92] Liang X., Jiang Z., Huang Z., Li F., Chen C., Hu C., Wang W., Hu Z., Liu Q., Wang B. (2020). Discovery of 6′-chloro-*N*-methyl-5′-(phenylsulfonamido)-[3,3′-bipyridine]-5-carboxamide (CHMFL-PI4K-127) as a novel *Plasmodium falciparum* PI(4)K inhibitor with potent antimalarial activity against both blood and liver stages of *Plasmodium*. Eur. J. Med. Chem..

[93] Le Roch K., Sestier C., Dorin D., Waters N., Kappes B., Chakrabarti D., Meijer L., Doerig C. (2000). Activation of a *Plasmodium falciparum* cdc2-related Kinase by Heterologous p25 and Cyclin H: Functionnal characterization of a *P. falciparum* Cyclin homologue. J. Biol. Chem..

[94] Ross-Macdonald P.B., Graeser R., Kappes B., Franklin R., Williamson D.H. (1994). Isolation and expression of a gene specifying a cdc2-like protein kinase from the human malaria parasite *Plasmodium falciparum*. Eur. J. Biochem..

[95] Eubanks A.L., Perkins M.M., Sylvester K., Ganley J.G., Posfai D., Sanschargrin P.C., Hong J., Sliz P., Derbyshire E.R. (2018). In silico Screening and Evaluation of *Plasmodium falciparum* Protein Kinase 5 (PK5) Inhibitors. ChemMedChem.

[96] Bracchi-Ricard V., Barik S., Delvecchio C., Doerig C., Chakrabarti R., Chakrabarti D. (2000). *Pf*PK6, a novel cyclin-dependent kinase/mitogen-activated protein kinase-related protein kinase from *Plasmodium falciparum*. Biochem. J..

[97] Dorin D., Semblat J.-P., Poullet P., Alano P., Goldring J.P.D., Whittle C., Patterson S., Chakrabarti D., Doerig C. (2004). *Pf*PK7, an atypical MEK-related protein kinase, reflects the absence of classical three-component MAPK pathways in the human malaria parasite *Plasmodium falciparum*: An atypical MEK-like enzyme in malarial parasites. Mol. Microbiol..

[98] Dorin-Semblat D., Sicard A., Doerig C., Ranford-Cartwright L., Doerig C. (2008). Disruption of the *Pf*PK7 Gene Impairs Schizogony and Sporogony in the Human Malaria Parasite *Plasmodium falciparum*. Eukaryot. Cell.

[99] Philip N., Haystead T.A. (2007). Characterization of a UBC13 kinase in *Plasmodium falciparum*. Proc. Natl. Acad. Sci. USA.

[100] Raphemot R., Eubanks A.L., Toro-Moreno M., Geiger R.A., Hughes P.F., Lu K.-Y., Haystead T.A.J., Derbyshire E.R. (2019). *Plasmodium* PK9 Inhibitors Promote Growth of Liver-Stage Parasites. Cell. Chem. Biol..

[101] Bouloc N., Large J.M., Smiljanic E., Whalley D., Ansell K.H., Edlin C.D., Bryans J.S. (2008). Synthesis and in vitro evaluation of imidazopyridazines as novel inhibitors of the malarial kinase *Pf*PK7. Bioorg. Med. Chem. Lett..

[102] Merckx A., Echalier A., Langford K., Sicard A., Langsley G., Joore J., Doerig C., Noble M., Endicott J. (2008). Structures of *P. falciparum* Protein Kinase 7 Identify an Activation Motif and Leads for Inhibitor Design. Structure.

[103] Klein M., Dinér P., Dorin-Semblat D., Doerig C., Grøtli M. (2009). Synthesis of 3-(1,2,3-triazol-1-yl)- and 3-(1,2,3-triazol-4-yl)-substituted pyrazolo[3,4-*d*]pyrimidin-4-amines via click chemistry: Potential inhibitors of the *Plasmodium falciparum*
*Pf*PK7 protein kinase. Org. Biomol. Chem..

[104] McRobert L., Taylor C.J., Deng W., Fivelman Q.L., Cummings R.M., Polley S.D., Billker O., Baker D.A. (2008). Gametogenesis in Malaria Parasites Is Mediated by the cGMP-Dependent Protein Kinase. PLoS Biol..

[105] Moon R.W., Taylor C.J., Bex C., Schepers R., Goulding D., Janse C.J., Waters A.P., Baker D.A., Billker O. (2009). A cyclic GMP signalling module that regulates gliding motility in a malaria parasite. PLoS Pathog..

[106] Taylor H.M., McRobert L., Grainger M., Sicard A., Dluzewski A.R., Hopp C.S., Holder A.A., Baker D.A. (2010). The malaria parasite cyclic GMP-dependent protein kinase plays a central role in blood-stage schizogony. Eukaryot. Cell.

[107] Brochet M., Collins M.O., Smith T.K., Thompson E., Sebastian S., Volkmann K., Schwach F., Chappell L., Gomes A.R., Berriman M. (2014). Phosphoinositide Metabolism Links cGMP-Dependent Protein Kinase G to Essential Ca2+ Signals at Key Decision Points in the Life Cycle of Malaria Parasites. PLoS Biol..

[108] Govindasamy K., Jebiwott S., Jaijyan D.K., Davidow A., Ojo K.K., Van Voorhis W.C., Brochet M., Billker O., Bhanot P. (2016). Invasion of hepatocytes by *Plasmodium* sporozoites requires cGMP-dependent protein kinase and calcium dependent protein kinase 4. Mol. Microbiol..

[109] Vanaerschot M., Murithi J.M., Pasaje C.F.A., Ghidelli-Disse S., Dwomoh L., Bird M., Spottiswoode N., Mittal N., Arendse L.B., Owen E.S. (2020). Inhibition of Resistance-Refractory *P. falciparum* Kinase PKG Delivers Prophylactic, Blood Stage, and Transmission-Blocking Antiplasmodial Activity. Cell. Chem. Biol..

[110] Baker D.A., Stewart L.B., Large J.M., Bowyer P.W., Ansell K.H., Jiménez-Díaz M.B., El Bakkouri M., Birchall K., Dechering K.J., Bouloc N.S. (2017). A potent series targeting the malarial cGMP-dependent protein kinase clears infection and blocks transmission. Nat. Commun..

[111] Biftu T., Feng D., Fisher M., Liang G.-B., Qian X., Scribner A., Dennis R., Lee S., Liberator P.A., Brown C. (2006). Synthesis and SAR studies of very potent imidazopyridine antiprotozoal agents. Bioorg. Med. Chem. Lett..

[112] Large J.M., Birchall K., Bouloc N.S., Merritt A.T., Smiljanic-Hurley E., Tsagris D.J., Wheldon M.C., Ansell K.H., Coombs P.J., Kettleborough C.A. (2019). Potent bicyclic inhibitors of malarial cGMP-dependent protein kinase: Approaches to combining improvements in cell potency, selectivity and structural novelty. Bioorg. Med. Chem. Lett..

[113] Large J.M., Birchall K., Bouloc N.S., Merritt A.T., Smiljanic-Hurley E., Tsagris D.J., Wheldon M.C., Ansell K.H., Coombs P.J., Kettleborough C.A. (2019). Potent inhibitors of malarial *P. Falciparum* protein kinase G: Improving the cell activity of a series of imidazopyridines. Bioorg. Med. Chem. Lett..

[114] Diaz C.A., Allocco J., Powles M.A., Yeung L., Donald R.G.K., Anderson J.W., Liberator P.A. (2006). Characterization of *Plasmodium falciparum* cGMP-dependent protein kinase (PfPKG): Antiparasitic activity of a PKG inhibitor. Mol. Biochem. Parasitol..

[115] Biftu T., Feng D., Ponpipom M., Girotra N., Liang G.-B., Qian X., Bugianesi R., Simeone J., Chang L., Gurnett A. (2005). Synthesis and SAR of 2,3-diarylpyrrole inhibitors of parasite cGMP-dependent protein kinase as novel anticoccidial agents. Bioorg. Med. Chem. Lett..

[116] Tsagris D.J., Birchall K., Bouloc N., Large J.M., Merritt A., Smiljanic-Hurley E., Wheldon M., Ansell K.H., Kettleborough C., Whalley D. (2018). Trisubstituted thiazoles as potent and selective inhibitors of *Plasmodium falciparum* protein kinase G (PfPKG). Bioorg. Med. Chem. Lett..

[117] Matralis A.N., Malik A., Penzo M., Moreno I., Almela M.J., Camino I., Crespo B., Saadeddin A., Ghidelli-Disse S., Rueda L. (2019). Development of Chemical Entities Endowed with Potent Fast-Killing Properties against *Plasmodium falciparum* Malaria Parasites. J. Med. Chem..

[118] Ul Mahmood S., Cheng H., Tummalapalli S.R., Chakrasali R., Yadav Bheemanaboina R.R., Kreiss T., Chojnowski A., Eck T., Siekierka J.J., Rotella D.P. (2020). Discovery of isoxazolyl-based inhibitors of *Plasmodium falciparum* cGMP-dependent protein kinase. RSC Med. Chem..

[119] Reyes P., Rathod P.K., Sanchez D.J., Mrema J.E.K., Rieckmann K.H., Heidrich H.-G. (1982). Enzymes of purine and pyrimidine metabolism from the human malaria parasite, *Plasmodium falciparum*. Mol. Biochem. Parasitol..

[120] Ashton T.D., Devine S.M., Möhrle J.J., Laleu B., Burrows J.N., Charman S.A., Creek D.J., Sleebs B.E. (2019). The Development Process for Discovery and Clinical Advancement of Modern Antimalarials. J. Med. Chem..

[121] Cui H., Carrero-Lerida J., Silva A.P.G., Whittingham J.L., Brannigan J.A., Ruiz-Perez L.M., Read K.D., Wilson K.S., Gonzalez-Pacanowska D., Gilbert I.H. (2012). Synthesis and Evaluation of α-Thymidine Analogs as Novel Antimalarials. J. Med. Chem..

[122] Kato A., Yasuda Y., Kitamura Y., Kandeel M., Kitade Y. (2012). Carbocyclic thymidine derivatives efficiently inhibit *Plasmodium falciparum* thymidylate kinase (PfTMK). Parasitol. Int..

[123] Noguchi Y., Yasuda Y., Tashiro M., Kataoka T., Kitamura Y., Kandeel M., Kitade Y. (2013). Synthesis of carbocyclic pyrimidine nucleosides and their inhibitory activities against *Plasmodium falciparum* thymidylate kinase. Parasitol. Int..

[124] Zhang M., Wang C., Otto T.D., Oberstaller J., Liao X., Adapa S.R., Udenze K., Bronner I.F., Casandra D., Mayho M. (2018). Uncovering the essential genes of the human malaria parasite *Plasmodium falciparum* by saturation mutagenesis. Science.

